# Supplementary material for: Tissue-infiltrating macrophages mediate an exosome-based metabolic reprogramming upon DNA damage
Source: Nat Commun. 2020 Jan 2;11:42. doi: 10.1038/s41467-019-13894-9 (PMC6940362; doi:10.1038/s41467-019-13894-9)
Supplement: Supplementary file 1 — Supplementary information [file 41467_2019_13894_MOESM1_ESM.pdf]

Supplementary information

Goulielmaki E. et al., Tissue-infiltrating macrophages mediate an exosome-based metabolic reprogramming upon DNA damage

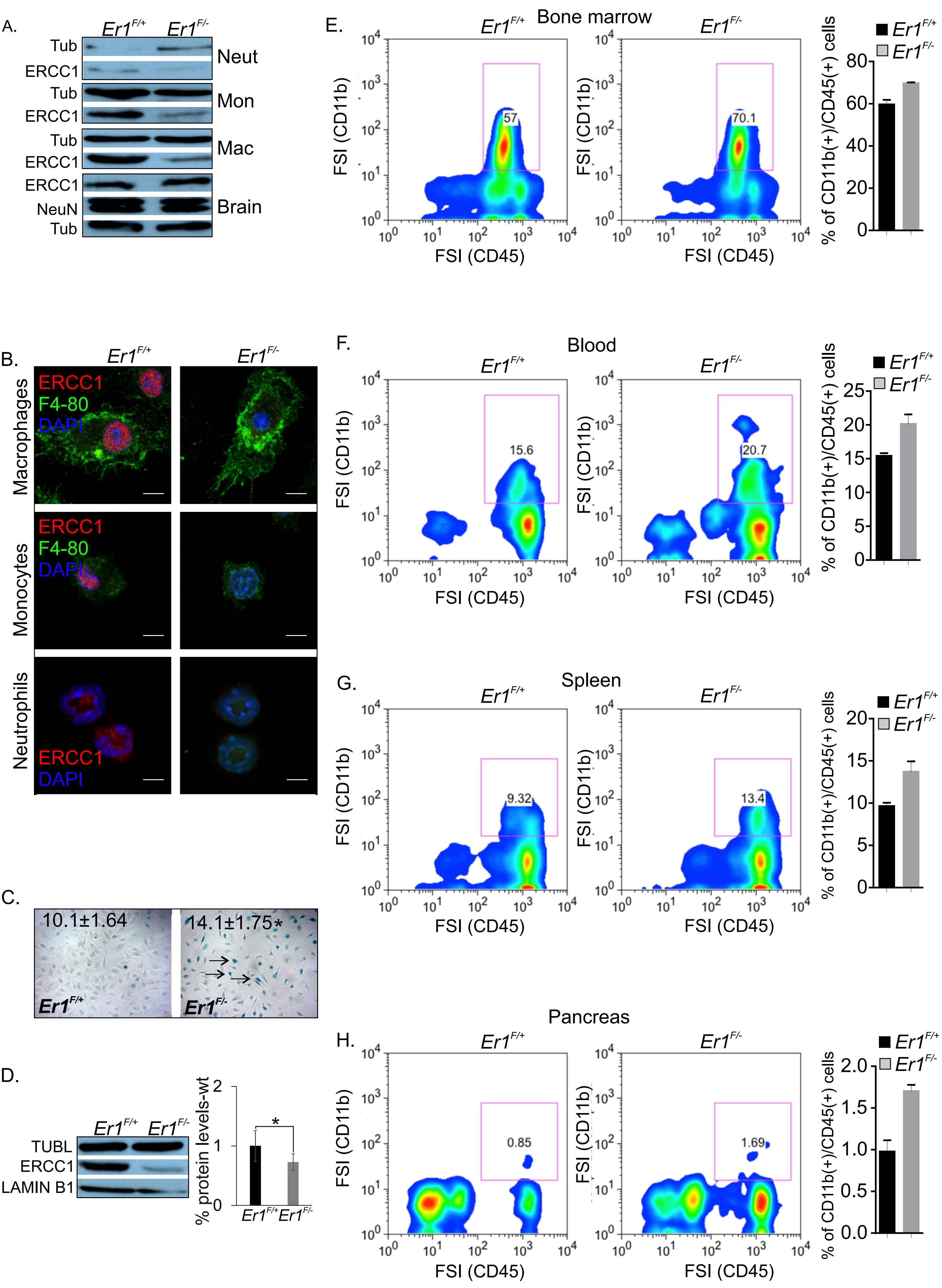

Supplementary Figure 1

Supplementary Figure 1: Expression of the Cre transgene in *Er1*$^{F/-}$ animals. (A). Western blotting of ERCC1 in neutrophils, monocytes, macrophages and brain tissue derived from *Er1*$^{F/-}$ and *Er1*$^{F/+}$ animals. (B). Immunofluorescence detection of ERCC1 and macrophage marker F4-80 in *Er1*$^{F/-}$ and *Er1*$^{F/+}$ macrophages, monocytes and neutrophils (as indicated). (C). SA-β-gal-staining in *Er1*$^{F/-}$ and *Er1*$^{F/+}$ BMDMs; n>500 cells were counted for each genotype from three independent experiments; Asterisk indicates the significance set at p-value: *≤0.05, one-tailed Student's t-test. (D). Western blotting of Lamin B1 in *Er1*$^{F/-}$ and *Er1*$^{F/+}$ BMDMs (n=5). Graphs represent data from Western blotting quantification. (E). FACS representative plots and respective graphs of CD45+CD11b+ cells, in the bone marrow, (F). blood, (G). spleen and (H). pancreas of 6-months old *Er1*$^{F/-}$ and *Er1*$^{F/+}$ animals (as indicated; n=6). All tissues are derived from the same *Er1*$^{F/-}$ and *Er1*$^{F/+}$ animals. Significance is set at P<0.05 (unpaired *t*-test). Asterisk indicates the significance set at p-value: *≤0.05, **≤0.01 (two-tailed Student's t-test unless stated otherwise). Grey line is set at 5μm scale.

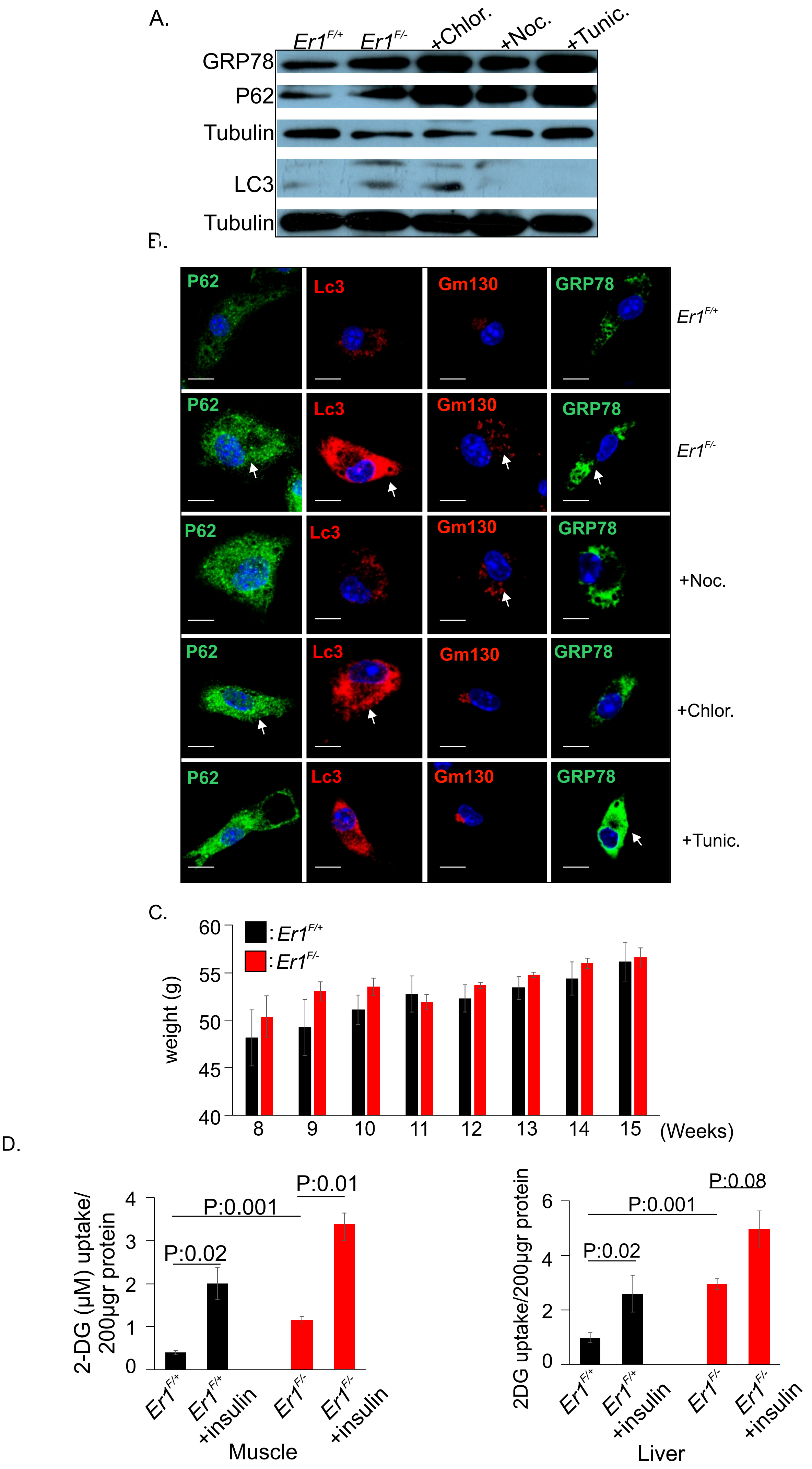

Supplementary Figure 2

Supplementary Figure 2: Cytoplasmic stress responses in *Er1*$^{F/-}$ macrophages and metabolic alterations in *Er1*$^{F/-}$ mice. (A). Western blotting of GRP78, P62, LC3 and Tubulin in *Er1*$^{F/-}$ and *Er1*$^{F/+}$ BMDMs and *Er1*$^{F/+}$ BMDMs treated with nocodazole (noc; known to trigger Golgi dispersal), chloroquine (Chlor.; known to inhibit the degradation of autophagosomes in lysosomes) and tunicamycin (Tunic.; known to trigger ER stress). (B). Confocal studies of P62, LC3, Gm130, and GRP78 in *Er1*$^{F/-}$ or *Er1*$^{F/+}$ BMDMs and *Er1*$^{F/+}$ BMDMs treated with nocodazole (noc.), chloroquine (Chlor.) and tunicamycin (Tunic.). (C). Weights of *Er1*$^{F/-}$ and *Er1*$^{F/+}$ animals (n=4) fed on a high fat diet for a period of 8-15 weeks (as indicated). (D). 2-Deoxy-D-glucose (2-DG) uptake (µM) in muscle and liver protein extracts derived from overnight starved *Er1*$^{F/-}$ and *Er1*$^{F/+}$ animals injected with insulin (as indicated). Error bars indicate S.E.M. among replicates ($n \geq 3$). Significance set at p-value: *≤0.05, **≤0.01 (two-tailed Student's t-test). Grey line is set at 5µm scale.

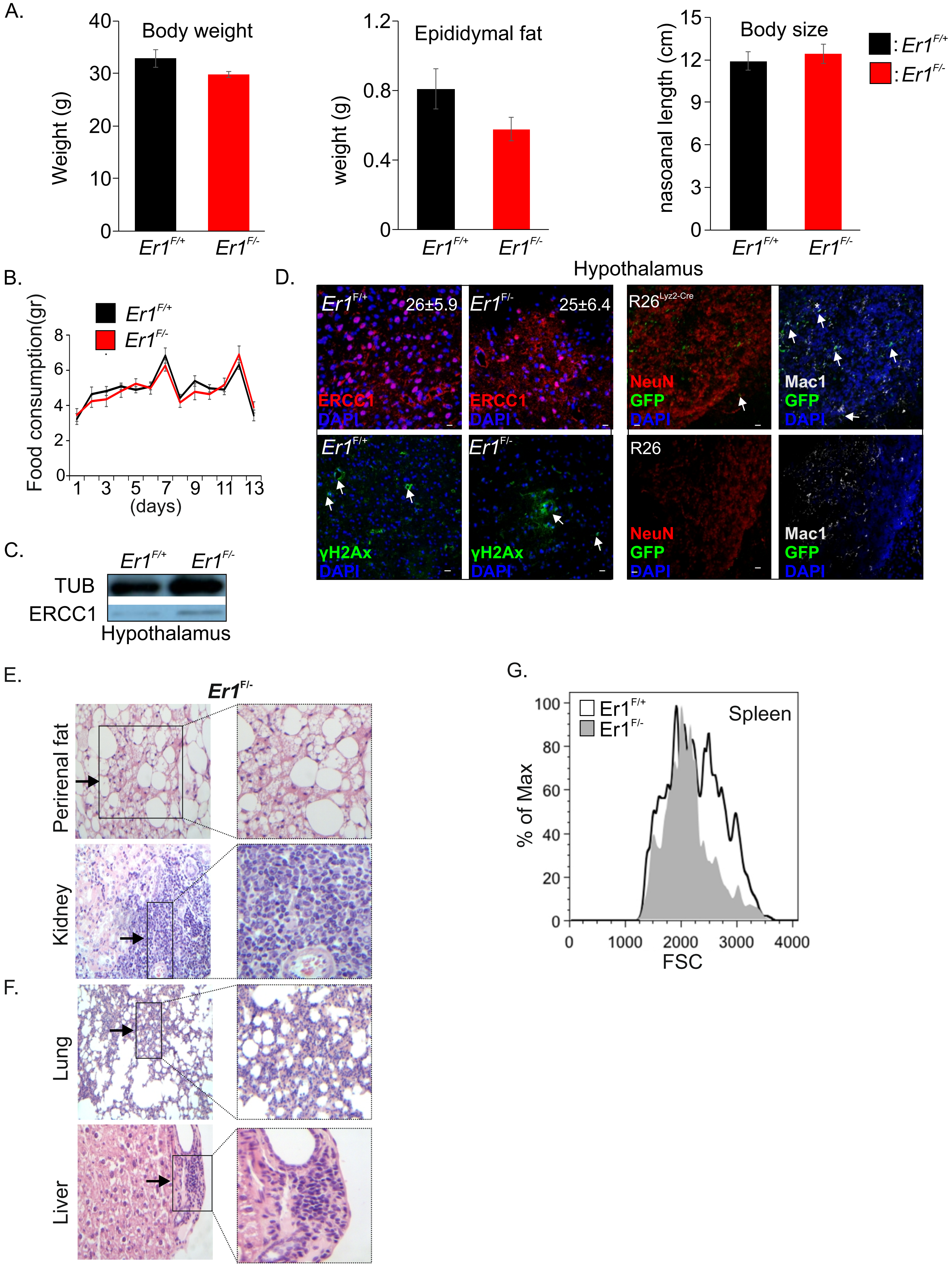
A.
Body weight
Weight (g)
Er1F/+
Er1F/-
Epididymal fat
weight (g)
Body size
nasoanal length (cm)
: Er1F/+
: Er1F/-
B.
Food consumption(gr)
(days)
C.
TUB
ERCC1
Hypothalamus
D.
Hypothalamus
26±5.9
25±6.4
ERCC1
DAPI
R26Lyz2-Cre
R26
NeuN
GFP
Mac1
γH2Ax
E.
Perirenal fat
Kidney
F.
Lung
Liver
G.
Spleen
% of Max
FSC

Supplementary Figure 3: Chronic inflammation in *Er1*$^{F/-}$ mice. (A). Body weights, weighs of epididymal fat depots and nasoanal length of 2-months old *Er1*$^{F/-}$ (n=10) and *Er1*$^{F/+}$ (n=11) animals. (B) Food intake (g) of 7-months old males *Er1*$^{F/-}$ and *Er1*$^{F/+}$ animals (n=7) over a period of 14 days. (C) Western blotting of ERCC1 in the hypothalamus of *Er1*$^{F/-}$ and *Er1*$^{F/+}$ animals. (D) Left panel: immunofluorescence detection of ERCC1 and γ-H2AX in *Er1*$^{F/-}$ and *Er1*$^{F/+}$ hypothalami. White colored numbers indicate the percentage of ERCC1(+) cells in *Er1*$^{F/-}$ and *Er1*$^{F/+}$ hypothalamic regions. Right panel: Lys-*Cre*-driven Rosa-YFP expression in the hypothalamus of *Er1*$^{F/-}$ and *Er1*$^{F/+}$animals. Co-staining with NeuN and MAC-1. (E). Magnified inlays showing the infiltration of foamy cells (macrophages) in a region of lipogranuloma in *Er1*$^{F/-}$ perirenal fat and lymphocyte and monocyte infiltrates in *Er1*$^{F/-}$ kidneys. Dashed box indicates magnified inlays. (F). Magnified inlays of the lymphocyte and monocyte infiltrates in *Er1*$^{F/-}$ lungs and livers. Dashed box indicates magnified inlays. (G). Representative forward scatter histograms of CD45+CD11b+ macrophages in the spleen of *Er1*$^{F/+}$ (grey-shaded peak) and *Er1*$^{F/-}$ (black-lined peak) mice (n=6) as measured by flow cytometry. The presence of a second FSC peak in *Er1*$^{F/-}$ histogram depicts the existence of a population of bone marrow derived monocytes/macrophages with higher volume, indicative of their activation/differentiation status. Grey line is set at 10μm scale.

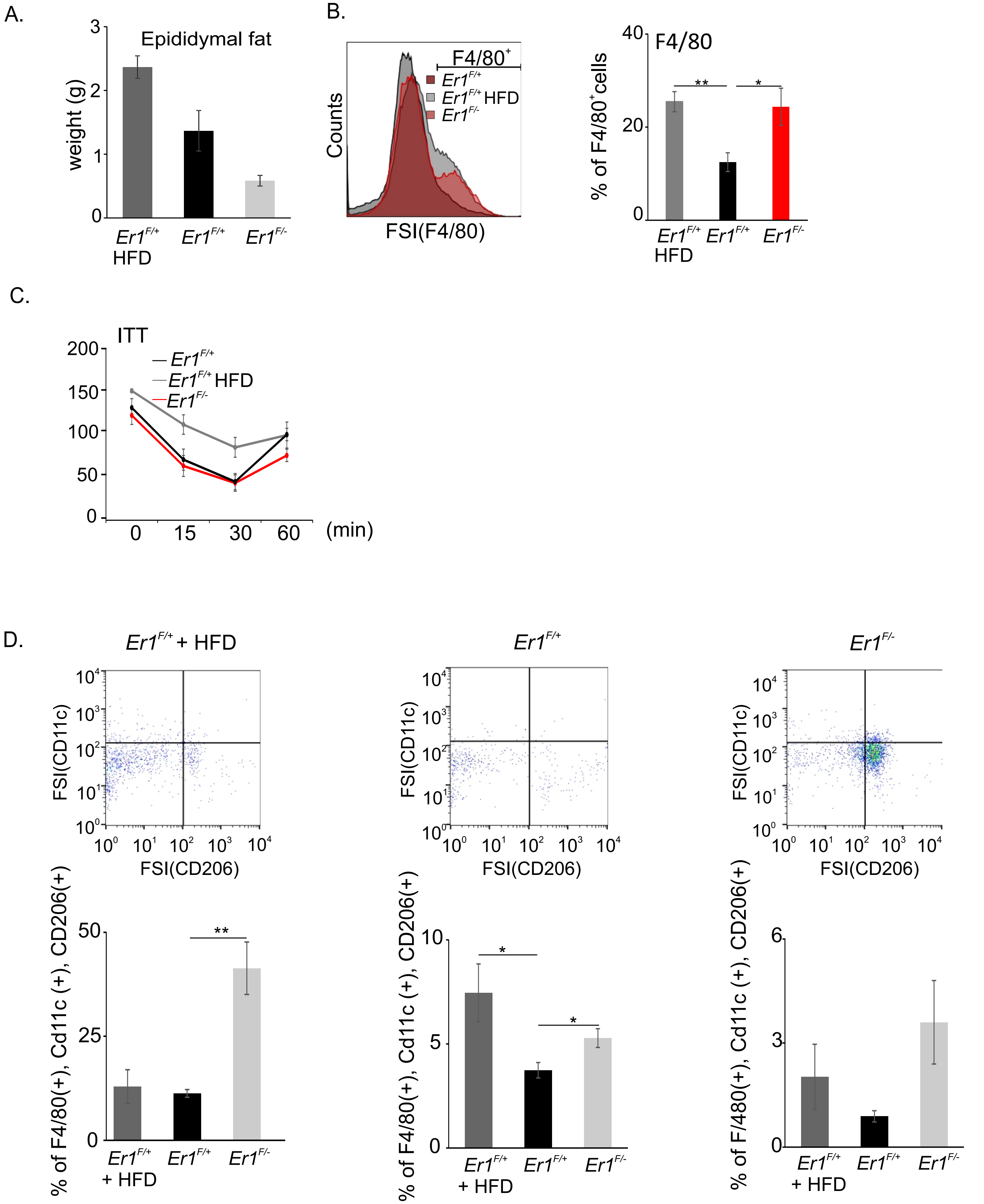

Supplementary Figure 4

Supplementary Figure 4: Characterization of adipose tissue macrophages in *Er1*$^{F/-}$ mice. (A) Weighs of epididymal fat depots of *Er1*$^{F/+}$, *Er1*$^{F/+}$ HFD and *Er1*$^{F/-}$ mice. (B) Representative histograms of F4/80^{+} macrophages in the epididymal fat of *Er1*$^{F/+}$, *Er1*$^{F/+}$ HFD and *Er1*$^{F/-}$ mice (n=3, 8-months old) as measured by flow cytometry (C) Insulin tolerance test (ITT) graphs of 8-months old *Er1*$^{F/+}$, *Er1*$^{F/+}$ HFD and *Er1*$^{F/-}$ mice. (D). FACS representative plots and respective graphs of F480+/CD11c^{+}, F480+/CD206^{+} and F480+/double cells, in the white adipose tissue of *Er1*$^{F/+}$, *Er1*$^{F/+}$ HFD and *Er1*$^{F/-}$ mice. Significance is set at P<0.05 (unpaired *t*-test). Asterisk indicates the significance set at p-value: *≤0.05, **≤0.01 (two-tailed Student's t-test).

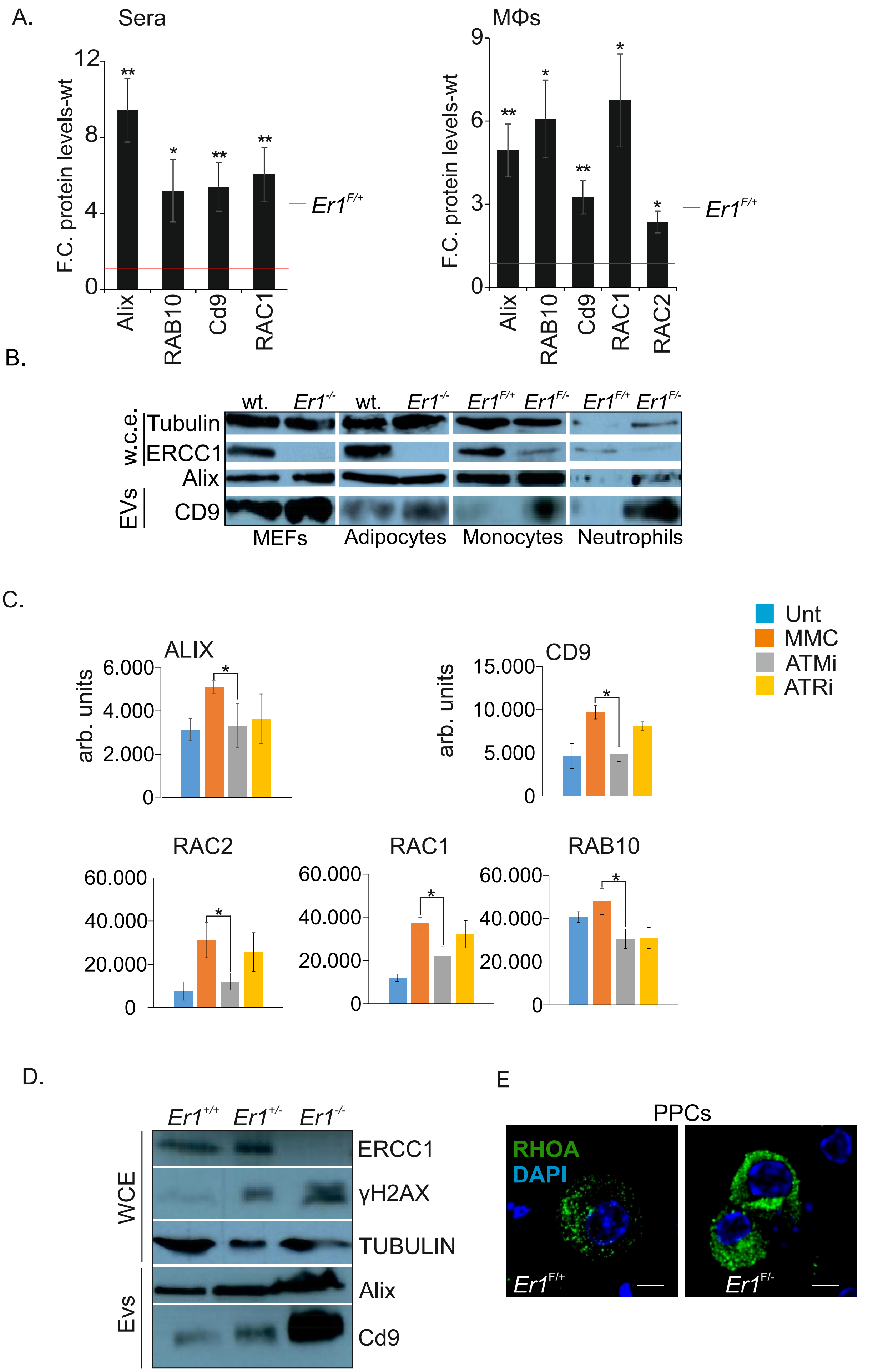

Supplementary Figure 5

Supplementary Figure 5: DNA damage triggers the release of EVs in *Er1*$^{F/-}$ macrophages. (A). The graph shows the western blot fold changes (F.C.; see Figure 5F) of the indicated proteins (x-axis) in the *Er1*$^{F/-}$ compared to *Er1*$^{F/+}$ sera (EV fraction, n=6; left panel) and in the EV fraction of *Er1*$^{F/-}$ macrophage culture media compared to the corresponding fraction of *Er1*$^{F/+}$ macrophage culture media (n=5; right panel; see Figure 5H) (B) Western blotting of ERCC1, Alix , CD9 and tubulin in the EV fraction or in whole cell extracts (w.c.e) of *Ercc1*$^{-/-}$ (*Er1*$^{-/-}$ and wt. MEFs or adipocytes and of *Er1*$^{F/-}$ or *Er1*$^{F/+}$ monocytes and neutrophils as indicated). (C). Quantitative measurement (arbitrary; arb. units) of ALIX, CD9, RAC2, RAC1 and RAB10 protein levels as measured in the EV fraction of MMC-treated and control BMDMs exposed to ATM (ATMi) or ATR (ATRi) inhibitors (n=3 independent experiments) (as indicated; see also Figure 5I. (D).Western blotting of γH2Ax, ERCC1 and tubulin in whole cell extracts (WCE) or ALIX and CD9 in the EV fraction of *Er1*$^{+/+}$, *Er1*$^{+/-}$ and *Er1*$^{-/-}$ BMDMs or culture media, respectively. (E). Immunofluorescence detection of RHOA in *Er1*$^{F/-}$ and *Er1*$^{F/+}$ primary pancreatic cells (PPCs). Error bars indicate S.E.M. ($n \geq 3$). Asterisk indicates the significance set at p-value: *≤0.05, **≤0.01 (two-tailed Student's t-test). Grey line is set at 5μm scale.

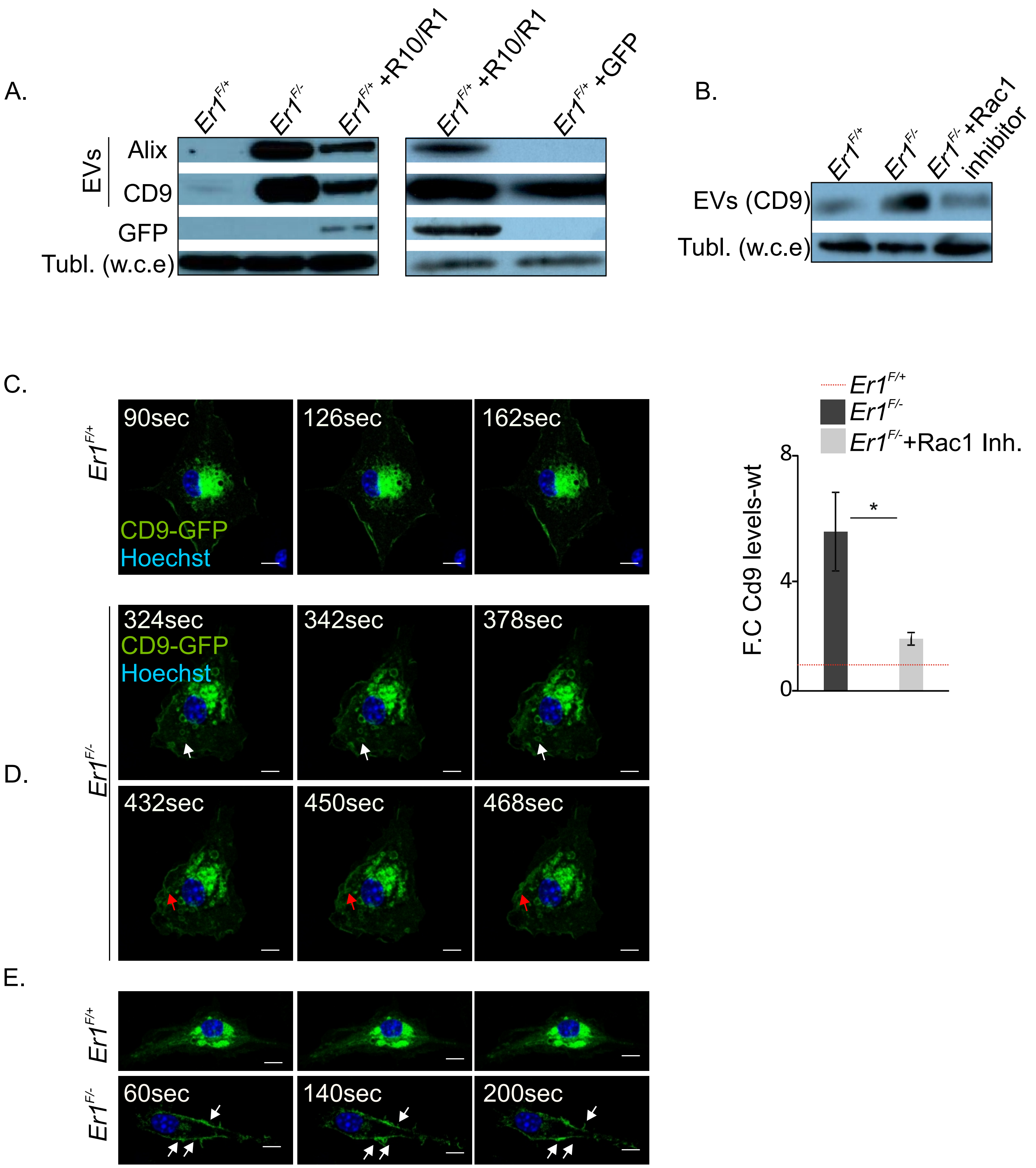

Supplementary Figure 6

Supplementary Figure 6. (A). Western blotting of Alix, CD9 and the GFP in EVs and tubulin (Tubl.) in whole cell extracts (w.c.e.) of *Er1*$^{F/-}$, *Er1*$^{F/+}$ and *Er1*$^{F/+}$ macrophages transfected with GFP-tagged RAC1 (R1) and GFP-tagged RAB10 (R10) or GFP alone (as indicated). (B). Western blotting of CD9 in the EV fraction and tubulin in whole cell extracts of *Er1*$^{F/+}$, *Er1*$^{F/-}$ and *Er1*$^{F/-}$ treated with NSC23766, 16hrs (50µM Rac1 inhibitor). Graph shows quantification of western blot (n=3). (C) Live confocal imaging of *Er1*$^{F/+}$ BMDMs and (D). *Er1*$^{F/-}$ BMDMs transiently expressing CD9-GFP. The images depict the static appearance of *Er1*$^{F/+}$ BMDM (upper panel; as indicated), the formation of a newly generated vesicle-like structure in the cytoplasm (white arrowhead) and a fusion event between a vesicle-like structure in the cytoplasm and the cell membrane (red arrowhead) in *Er1*$^{F/-}$ BMDMs. (E). Live confocal imaging of *Er1*$^{F/+}$ BMDMs and *Er1*$^{F/-}$ BMDMs transiently expressing CD9-GFP. The images depict the static appearance of *Er1*$^{F/+}$ BMDM (upper panel; as indicated) and the progressive formation of a protruded pseudopodium in the membrane of *Er1*$^{F/-}$ BMDMs. Images are generated every 18-20 sec for 10-12 minutes yielding a total of 20-30 frames per movie. Images are representative of a total of at least 20 cells per genotype recorded in 4 independent experiments. Grey line is set at 5µm scale.

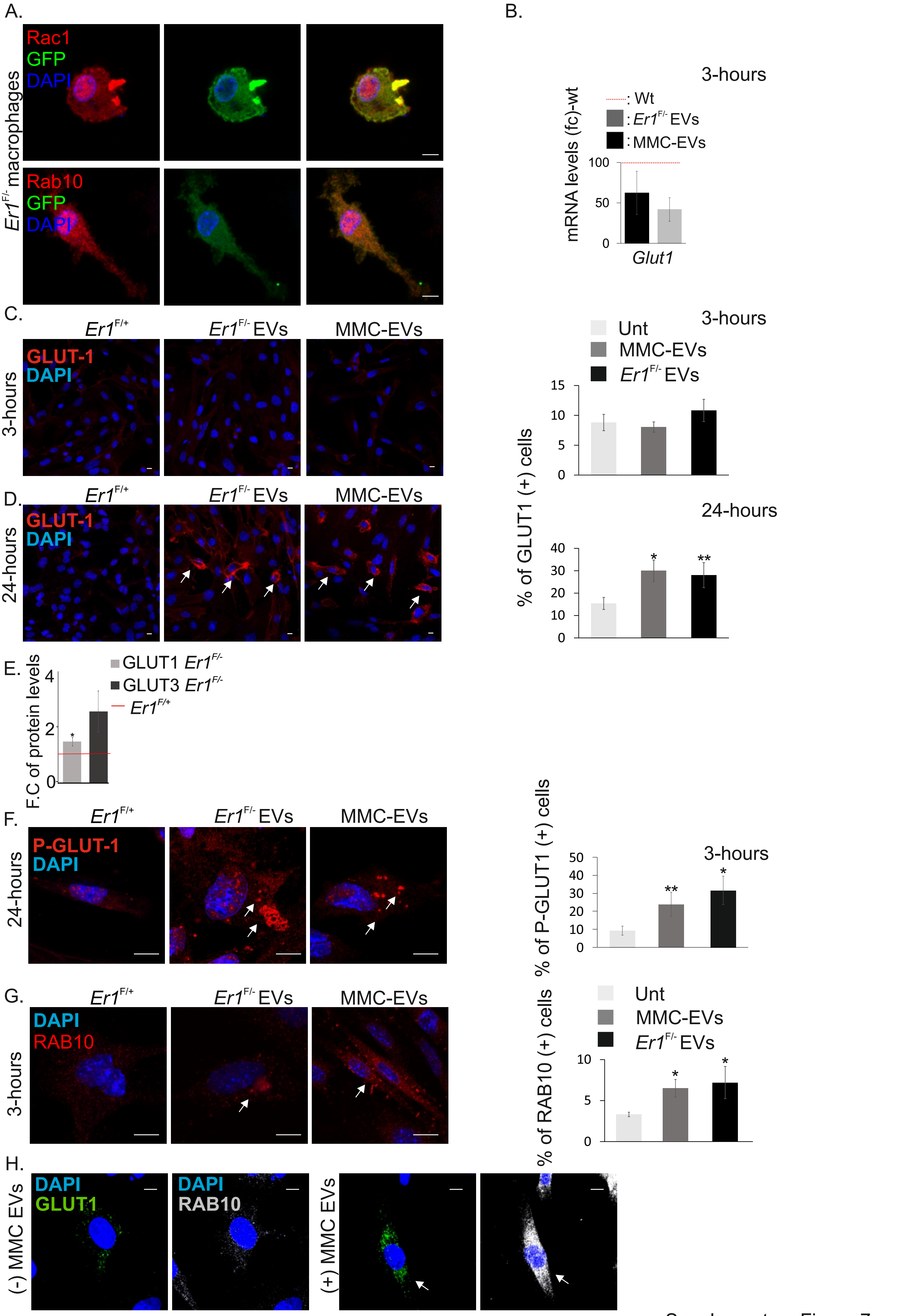

Supplementary Figure 7

Supplementary Figure 7. *Er1*$^{F/-}$-derived exosomes stimulate the glucose uptake in targeted cells. (A). Immunofluorescence detection of GFP-tagged RAC1 and GFP-tagged RAB10 in *Er1*$^{F/-}$ macrophages. (B*). Glut1* mRNA levels (as fold change; fc) in PPCs exposed to EVs derived from *Er1*$^{F/-}$ (*Er1*$^{F/-}$ EVs) or MMC-treated (MMC-EVs) macrophages compared to untreated wt. macrophages (red dotted line) for 3 hrs. (C). Immunofluorescence detection of GLUT1 in PPCs exposed to EVs derived from *Er1*$^{F+}$ (*Er1*$^{F+}$ EVs), *Er1*$^{F/-}$ (*Er1*$^{F/-}$ EVs) or MMC-treated EVs (MMC-EVs) for 3- or (D). 24hours (as indicated). (E). The graph shows the fold change of GLUT1 and GLUT3 protein levels in primary pancreatic cells exposed to *Er1*$^{F/-}$ or *Er1*$^{F+}$ EVs (n=4; the data accompany the western blots shown in Figure 6G (F). Immunofluorescence detection of P-GLUT1 in PPCs exposed to *Er1*$^{F+}$ EVs, *Er1*$^{F/-}$ EVs or to MMC-EVs for 24hours. (G). Immunofluorescence detection of RAB10 in PPCs exposed to *Er1*$^{F+}$ EVs, *Er1*$^{F/-}$ EVs or to MMC-EVs for 3 hours. (H). Immunofluorescence detection of GLUT1 and RAB10 in PPCs exposed to MMC-EVs for 24hours. The graphs show the % of positively stained cells per condition (n> 200 cells were counted in at least 5 optical fields). Error bars indicate S.E.M. ($n \geq 3$). Asterisk indicates the significance set at p-value: *≤0.05, **≤0.01 (two-tailed Student's t-test). Grey line is set at 5μm scale.

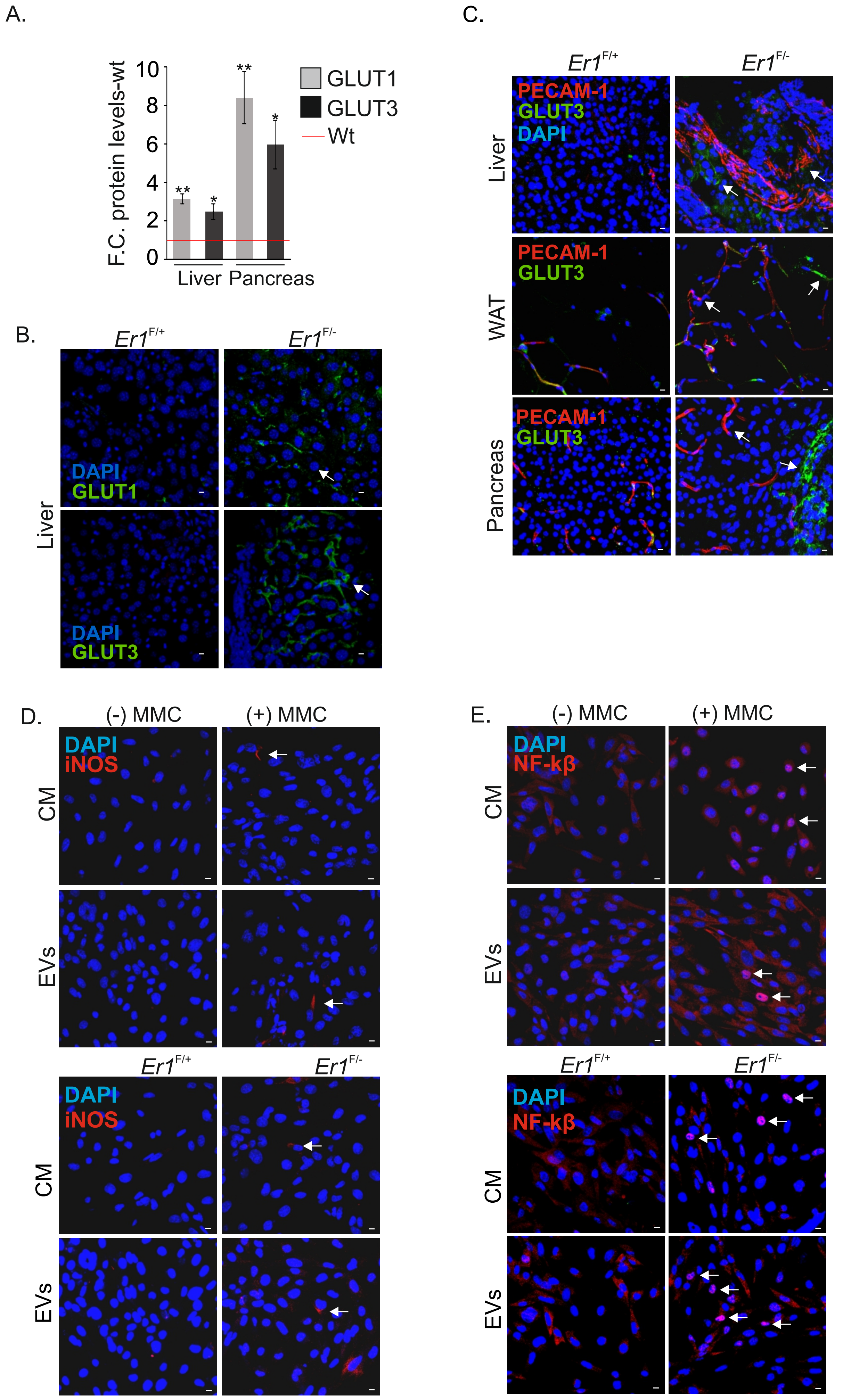
A.
F.C. protein levels-wt
10
8
6
4
2
0
**
*
**
*
Liver
Pancreas
GLUT1
GLUT3
Wt
B.
Er1F/+
Er1F/-
Liver
DAPI
GLUT1
DAPI
GLUT3
C.
Er1F/+
Er1F/-
Liver
PECAM-1
GLUT3
DAPI
WAT
PECAM-1
GLUT3
Pancreas
PECAM-1
GLUT3
D.
(-) MMC
(+) MMC
DAPI
iNOS
CM
EVs
Er1F/+
Er1F/-
DAPI
iNOS
CM
EVs
E.
(-) MMC
(+) MMC
DAPI
NF-kβ
CM
EVs
Er1F/+
Er1F/-
DAPI
NF-kβ
CM
EVs

Supplementary Figure 8. BMDM-derived glucose uptake triggers iNOS accumulation in a rapamycin-dependent manner. (A). The graph shows the fold change (F.C) of GLUT1 and GLUT3 protein levels in *Er1*$^{F/-}$ compared to *Er1*$^{F/+}$ livers and in *Er1*$^{F/-}$ compared to *Er1*$^{F/+}$ pancreata (n = 3; the data accompany Figure 6K). (B) Immunofluorescence detection of GLUT1 and GLUT3 in *Er1*$^{F/-}$ and *Er1*$^{F/+}$ livers. Grey line is set at 10 scale. (C). Immunofluorescence detection of PECAM-1 and GLUT3 (shown by the arrowhead) in *Er1*$^{F/-}$ and *Er1*$^{F/+}$ livers, white adipose tissue (WAT) and pancreata. Grey line is set at 10 scale. (D). Immunofluorescence detection of iNOS in PPCs exposed to i. culture media (CM) devoid of EVs or ii. only the EVs derived from MMC treated (MMC+) and untreated (MMC-) (upper panel) or *Er1*$^{F/-}$ and *Er1*$^{F/+}$ (lower panel) BMDMs (see also Figure 6A-B). (E). Immunofluorescence detection of NF-kβ in PPCs exposed to i. culture media (CM) devoid of EVs or ii. only the EVs derived from MMC treated (MMC+) and untreated (MMC-) (upper panel) or *Er1*$^{F/-}$ and *Er1*$^{F/+}$ (lower panel) BMDMs (see also Figure 6C-D). Grey line is set at 5μm scale.

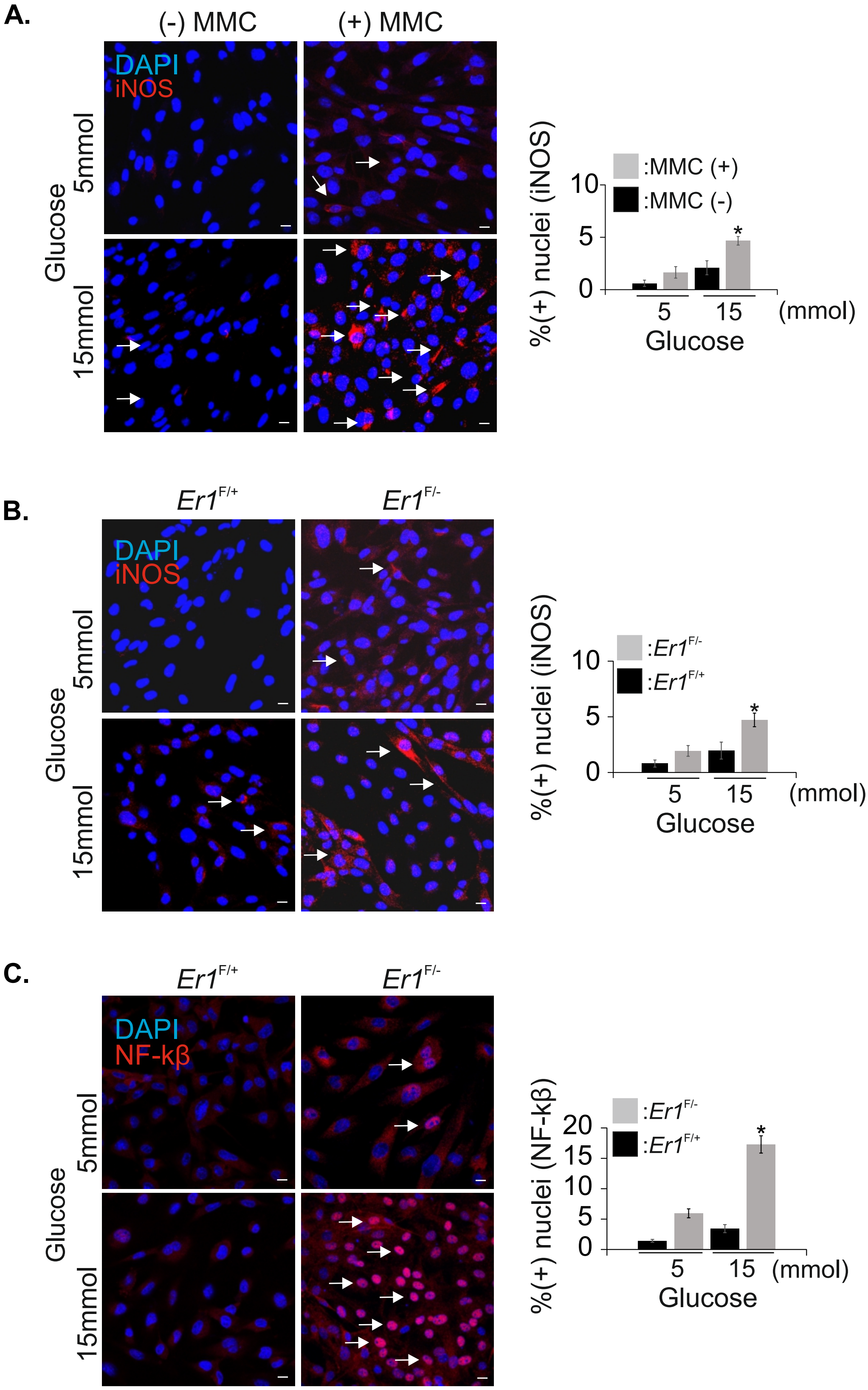

Supplementary Figure 9

Supplementary Figure 9. BMDM-derived glucose uptake triggers NF-kβ nuclear translocation in a rapamycin-dependent manner. (A). Immunofluorescence detection of iNOS in PPCs exposed to culture media (CM) and EVs derived from MMC-treated and untreated BMDMs or from (B). *Er1*$^{F/-}$ and *Er1*$^{F/+-}$BMDMs upon low (5mmol) and high (15mmol) glucose concentration. (C). Immunofluorescence detection of NF-kβ in PPCs exposed to CM) and EVs derived from *Er1*$^{F/-}$ and *Er1*$^{F+-}$BMDMs upon low (5mmol) and high (15mmol) glucose concentration. The graphs show the % of positively stained cells per condition (>200 cells were counted in at least 5 optical fields in each condition). Error bars indicate S.E.M. ($n \geq 3$). Asterisk indicates the significance set at p-value: *≤0.05, **≤0.01 (two-tailed Student's t-test). Grey line is set at 5μm scale.

A.

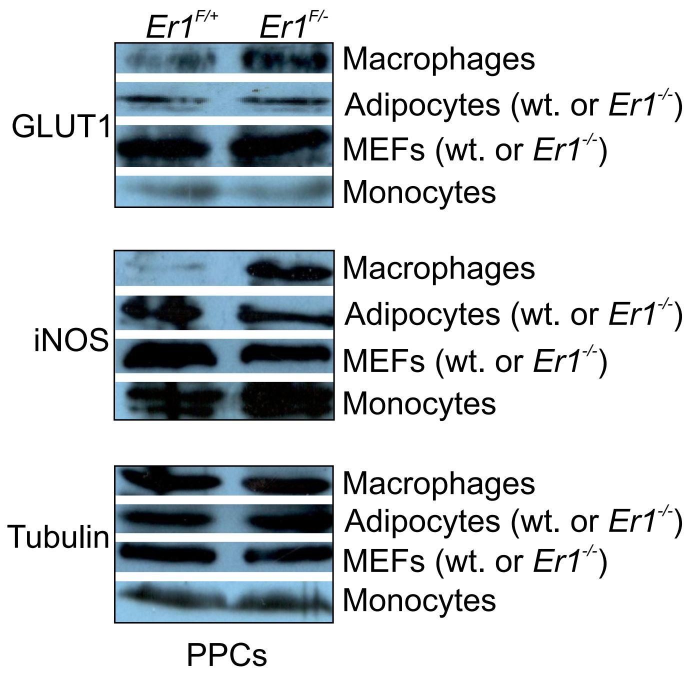
Er1F/+
Er1F/-
GLUT1
iNOS
Tubulin
Macrophages
Adipocytes (wt. or Er1-/-)
MEFs (wt. or Er1-/-)
Monocytes
PPCs

B.

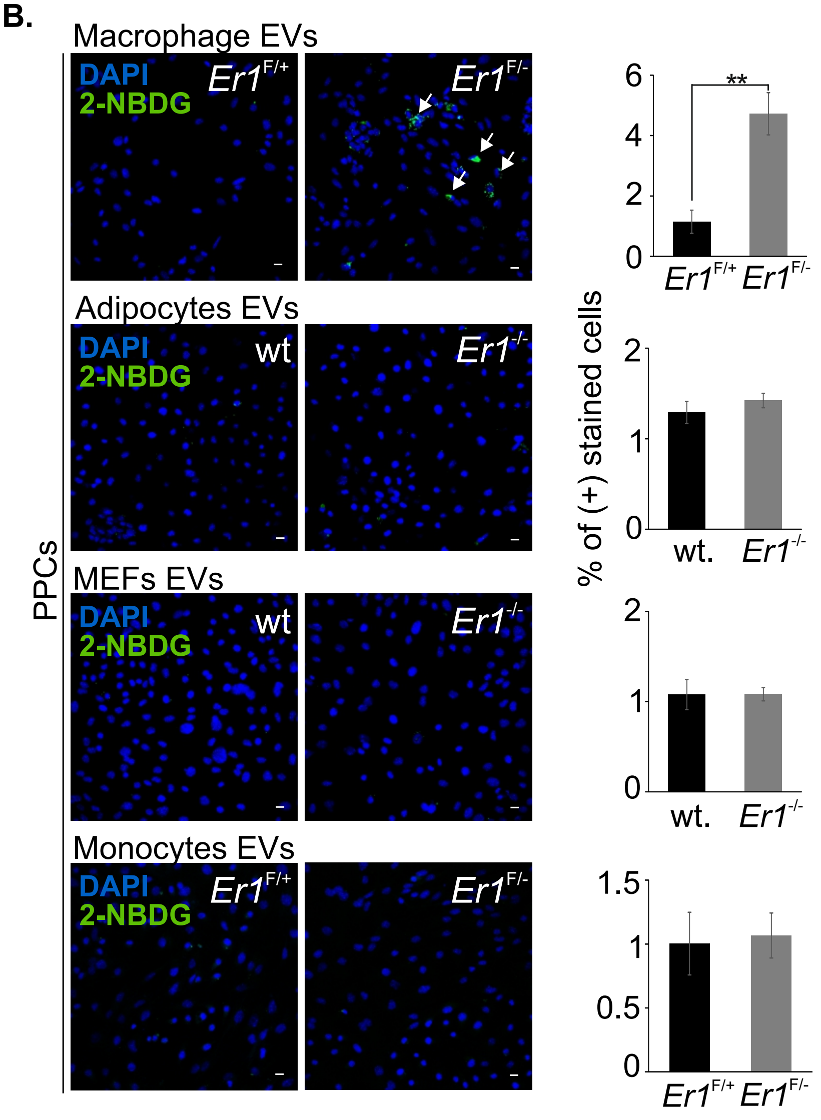
Macrophage EVs
Adipocytes EVs
MEFs EVs
Monocytes EVs
PPCs
DAPI
2-NBDG
Er1F/+
Er1F/-
wt
Er1-/-
**
% of (+) stained cells
wt.
6
4
2
0
1
1.5
0.5

Supplementary Figure 10. Cell type specificity of *Er1*$^{F/-}$ EV-driven glucose uptake and inflammation (A). Western blotting of GLUT1, iNOS and Tubulin in PPCs exposed to EVs derived from *Er1*$^{F/-}$ and *Er1*$^{F/+}$ macrophages or monocytes and from *Ercc1*$^{-/-}$ (*Er1*$^{-/-}$) and wt. adipocytes or MEFs (as indicated). (B). Immunofluorescence detection of 2-NBDG in PPCs exposed to EVs derived from *Er1*$^{F/-}$ or *Er1*$^{F/+}$ macrophages or monocytes and from *Ercc1*$^{-/-}$ (*Er1*$^{-/-}$) and wt. MEFs or adipocytes (as indicated). The graphs show the percentage of positively stained cells per condition (>500 cells were counted in at least 5 optical fields in each condition). Error bars indicate S.E.M. ($n \geq 3$). Asterisk indicates the significance set at p-value: **≤0.01 (two-tailed Student's t-test). Grey line is set at 5µm scale.
