# Supplementary material for: Tissue-infiltrating macrophages mediate an exosome-based metabolic reprogramming upon DNA damage
Source: Nat Commun. 2020 Jan 2;11:42. doi: 10.1038/s41467-019-13894-9 (PMC6940362; doi:10.1038/s41467-019-13894-9)
Supplement: Supplementary file 3 — Description of additional supplementary files [file 41467_2019_13894_MOESM3_ESM.docx]

**Description of Additional Supplementary Files**

**File Name**: Supplementary Movie 1
**Description:** Live confocal imaging of Er1F/+ BMDMs transiently expressing CD9-GFP. The movie depicts the sedentary appearance of Er1F/+ BMDMs.

**File Name:** Supplementary Movie 2
**Description:** Live confocal imaging of Er1F/+ BMDMs transiently expressing CD9-GFP. The movie depicts the sedentary appearance of Er1F/+ BMDMs.

**File Name:** Supplementary Movie 3
**Description:** Live confocal imaging of Er1F/- BMDMs transiently expressing CD9- GFP. The movie depicts the formation of a newly generated vesicle-like structure in the cytoplasm (white arrowhead) and a fusion event between a vesicle-like structure in the cytoplasm and the cell membrane (red arrowhead) in Er1F/- BMDMs.

**File Name**: Supplementary Movie 4
**Description:** Live confocal imaging of Er1F/- BMDMs transiently expressing CD9-GFP. The movie depicts the gradual appearance of pseudopodia in the cell membrane (white arrowhead) of Er1F/- BMDMs.

**File Name**: Supplementary Data 1

**Description:** Significant gene expression changes (RNA-Seq) in Er1F/- macrophages compared to Er1F/+ macrophages; FC: Fold change; FDR (False detection rate).

**File Name**: Supplementary Data 2

**Description:** Overview of the 211 shared proteins identified through a high-throughput mass spectrometry approach in Er1F/-macrophage media compared to corresponding Er1F/+ controls.
