# Supplementary material for: Tissue-infiltrating macrophages mediate an exosome-based metabolic reprogramming upon DNA damage
Source: Nat Commun. 2020 Jan 2;11:42. doi: 10.1038/s41467-019-13894-9 (PMC6940362; doi:10.1038/s41467-019-13894-9)
Supplement: Supplementary file 4 — Supplementary data1 [file 41467_2019_13894_MOESM4_ESM.pdf]

Supplementary data 1. Significant gene expression (RNA-seq) changes in ER F/- macrophages compared to ER F/+ macrophages. FC: Fold change; FDR: False detection rate.

Gene ID	Gene name	Meta-FDR	FC
ENSMUSG00000023039	Krt7	0,000000	33,60
ENSMUSG00000091345	Col6a5	0,000000	21,77
ENSMUSG00000031517	Gpm6a	0,000000	21,25
ENSMUSG00000042985	Upk3b	0,000000	19,50
ENSMUSG00000031343	Gabra3	0,000000	15,80
ENSMUSG00000029211	Gabra4	0,000000	15,73
ENSMUSG00000020607	Fam84a	0,000000	14,95
ENSMUSG00000020427	Igfbp3	0,000000	14,74
ENSMUSG00000057967	Fgf18	0,000000	14,38
ENSMUSG00000043719	Col6a6	0,000000	13,56
ENSMUSG00000046844	Vat1l	0,000000	13,28
ENSMUSG00000028989	Angptl7	0,000000	13,20
ENSMUSG00000039323	Igfbp2	0,000000	12,35
ENSMUSG00000021390	Ogn	0,000000	12,10
ENSMUSG00000027070	Lrp2	0,000000	11,74
ENSMUSG00000040703	Cyp2s1	0,000000	11,46
ENSMUSG00000053279	Aldh1a1	0,000000	11,33
ENSMUSG00000038725	Pkhd1l1	0,000000	11,25
ENSMUSG00000055027	Smyd1	0,000002	10,63
ENSMUSG00000049281	Scn3b	0,000000	10,53
ENSMUSG00000049436	Upk1b	0,000000	10,46
ENSMUSG00000046733	Gprc5a	0,000000	10,30
ENSMUSG00000026840	Lamc3	0,000000	10,24
ENSMUSG00000001739	Cldn15	0,000000	10,03
ENSMUSG00000019359	Gdpd2	0,000000	9,88
ENSMUSG00000031380	Vegfd	0,000000	9,85
ENSMUSG00000043110	Lrrn4	0,000000	9,80
ENSMUSG00000028871	Rspo1	0,000000	9,79
ENSMUSG00000029168	Dpysl5	0,000000	9,65
ENSMUSG00000022766	Serpind1	0,000048	9,57
ENSMUSG00000023046	Igfbp6	0,000000	9,57
ENSMUSG00000039457	Ppl	0,000002	9,56
ENSMUSG00000048387	Osr1	0,000000	9,47
ENSMUSG00000031906	Smpd3	0,000000	9,46
ENSMUSG00000037440	Vnn1	0,000017	9,31
ENSMUSG00000043613	Mmp3	0,000000	8,96
ENSMUSG00000021806	Nid2	0,000000	8,95
ENSMUSG00000004814	Ccl24	0,000000	8,88
ENSMUSG00000050052	Tdrp	0,000000	8,55
ENSMUSG00000022894	Adamts5	0,000000	8,47
ENSMUSG00000027820	Mme	0,000001	8,18
ENSMUSG00000037169	Mycn	0,000001	8,15

ENSMUSG00000020911	Krt19	0,000000	8,08
ENSMUSG00000058743	Kcnj14	0,000000	7,65
ENSMUSG00000056328	Myh1	0,000007	7,64
ENSMUSG00000052353	Cemip	0,000000	7,60
ENSMUSG00000029778	Adcyap1r1	0,000000	7,48
ENSMUSG00000021367	Edn1	0,000000	7,42
ENSMUSG00000055653	Gpc3	0,000000	7,40
ENSMUSG00000020467	Efemp1	0,000000	7,39
ENSMUSG00000010086	Rnf112	0,000003	7,38
ENSMUSG00000050926	Dcaf12l2	0,000659	7,36
ENSMUSG00000044177	Wfikkn2	0,000000	7,35
ENSMUSG00000025608	Podxl	0,000000	7,31
ENSMUSG00000031351	Zfp185	0,000004	7,29
ENSMUSG00000044217	Aqp5	0,000000	7,26
ENSMUSG00000026442	Nfasc	0,000000	7,22
ENSMUSG00000016494	Cd34	0,000000	7,14
ENSMUSG00000044017	Adgrd1	0,000000	7,14
ENSMUSG00000045284	Dcaf12l1	0,000006	7,12
ENSMUSG00000043259	Fam13c	0,000000	7,10
ENSMUSG00000041556	Fbxo2	0,000000	7,09
ENSMUSG00000031661	Nkd1	0,000000	7,06
ENSMUSG00000030669	Calca	0,000001	7,03
ENSMUSG00000057751	Megf6	0,000000	6,93
ENSMUSG00000021087	Rtn1	0,000000	6,87
ENSMUSG00000045954	Sdpr	0,000000	6,80
ENSMUSG00000027574	Nkain4	0,000000	6,78
ENSMUSG00000042757	Tmem108	0,000000	6,74
ENSMUSG00000055026	Gabrg3	0,000086	6,71
ENSMUSG00000027499	Pkia	0,000000	6,71
ENSMUSG00000001864	Aif1l	0,000000	6,70
ENSMUSG00000023963	Cyp39a1	0,000000	6,50
ENSMUSG00000036136	Fam110c	0,000000	6,43
ENSMUSG00000034881	Tbxa2r	0,000000	6,41
ENSMUSG00000033768	Nrxn2	0,000000	6,30
ENSMUSG00000025780	Itih5	0,000000	6,26
ENSMUSG00000029371	Cxcl5	0,000000	6,22
ENSMUSG00000063873	Slc24a3	0,000000	6,21
ENSMUSG00000036596	Cpz	0,000000	6,21
ENSMUSG00000024866	Acy3	0,000000	6,17
ENSMUSG00000048482	Bdnf	0,000068	6,14
ENSMUSG00000033361	Prrg3	0,000000	6,04
ENSMUSG00000034687	Fras1	0,000030	6,04
ENSMUSG00000019856	Fam184a	0,000896	6,00
ENSMUSG00000049404	Rarres1	0,000000	5,96
ENSMUSG00000062591	Tubb4a	0,000000	5,96
ENSMUSG00000022464	Slc38a4	0,000001	5,91
ENSMUSG00000040254	Sema3d	0,000061	5,89

ENSMUSG00000032338	Hcn4	0,000797	5,82
ENSMUSG00000019880	Rspo3	0,000009	5,80
ENSMUSG00000023918	Adgrf4	0,000026	5,76
ENSMUSG00000046694	Fam46b	0,000000	5,64
ENSMUSG00000061126	Cyp4f39	0,000010	5,64
ENSMUSG00000019647	Sema6a	0,000096	5,63
ENSMUSG00000048960	Prex2	0,000409	5,59
ENSMUSG00000015852	Fcrls	0,000064	5,50
ENSMUSG00000032006	Pdgfd	0,000003	5,50
ENSMUSG00000037206	Islr	0,000000	5,49
ENSMUSG00000068696	Gpr88	0,000006	5,48
ENSMUSG00000030513	Pcsk6	0,000067	5,44
ENSMUSG00000052957	Gas1	0,000000	5,44
ENSMUSG00000048040	Arxes2	0,000000	5,41
ENSMUSG00000049115	Agtr1a	0,000876	5,41
ENSMUSG00000021381	Barx1	0,000000	5,38
ENSMUSG00000056174	Col8a2	0,000000	5,36
ENSMUSG00000020684	Rasl10b	0,000006	5,36
ENSMUSG00000051022	Hs3st1	0,000001	5,34
ENSMUSG00000045180	Shroom2	0,000000	5,32
ENSMUSG00000048355	Arxes1	0,000048	5,27
ENSMUSG00000022865	Cxadr	0,000000	5,25
ENSMUSG00000073758	Sh3d21	0,000286	5,21
ENSMUSG00000024678	Ms4a4d	0,000014	5,17
ENSMUSG00000020431	Adcy1	0,000001	5,13
ENSMUSG00000042109	Csdc2	0,000013	5,11
ENSMUSG00000012428	Steap4	0,000000	5,11
ENSMUSG00000036412	Arsi	0,000002	5,09
ENSMUSG00000028776	Tinagl1	0,000000	5,08
ENSMUSG00000026043	Col3a1	0,000000	5,07
ENSMUSG00000023078	Cxcl13	0,000000	5,07
ENSMUSG00000036782	Klhl13	0,000000	5,06
ENSMUSG00000032348	Gsta4	0,000028	5,06
ENSMUSG00000039396	Neil3	0,000000	5,05
ENSMUSG00000015085	Entpd2	0,000000	5,04
ENSMUSG00000020871	Dlx4	0,000393	5,04
ENSMUSG00000048772	Tmem53	0,000019	5,00
ENSMUSG00000037157	Il22ra1	0,000000	5,00
ENSMUSG00000034936	Arl4d	0,000000	4,99
ENSMUSG00000040612	Ildr2	0,000000	4,99
ENSMUSG00000044641	Pard6b	0,000000	4,99
ENSMUSG00000015627	Gata5	0,000000	4,97
ENSMUSG00000038486	Sv2a	0,000000	4,95
ENSMUSG00000032839	Trpc1	0,002769	4,95
ENSMUSG00000035403	Crb2	0,000000	4,93
ENSMUSG00000001334	Fndc5	0,000012	4,92
ENSMUSG00000042367	Gjb3	0,000000	4,91

ENSMUSG00000030849	Fgfr2	0,000000	4,90
ENSMUSG00000027996	Sfrp2	0,000000	4,88
ENSMUSG00000017692	Rhbdl3	0,000000	4,87
ENSMUSG00000024481	Lvrn	0,000436	4,86
ENSMUSG00000090084	Srpx	0,000000	4,86
ENSMUSG00000067276	Capn6	0,000036	4,84
ENSMUSG00000056870	Gulp1	0,000001	4,84
ENSMUSG00000044749	Abca6	0,000146	4,83
ENSMUSG00000036356	Csgalnact1	0,000035	4,83
ENSMUSG00000025105	Bnc1	0,000000	4,80
ENSMUSG00000029999	Tgfa	0,000002	4,79
ENSMUSG00000047420	Fam180a	0,000004	4,79
ENSMUSG00000036446	Lum	0,000021	4,75
ENSMUSG00000063415	Cyp26b1	0,000092	4,74
ENSMUSG00000000126	Wnt9a	0,000000	4,74
ENSMUSG00000033910	Gucy1a3	0,000003	4,70
ENSMUSG00000027896	Slc16a4	0,000000	4,68
ENSMUSG00000022665	Ccdc80	0,000000	4,66
ENSMUSG00000044788	Fads6	0,000000	4,65
ENSMUSG00000004044	Ptrf	0,000000	4,63
ENSMUSG00000048332	Lhfp	0,000000	4,62
ENSMUSG00000035413	Tmem98	0,000000	4,62
ENSMUSG00000024803	Ankrd1	0,000000	4,57
ENSMUSG00000079164	Tlr5	0,000235	4,57
ENSMUSG00000021057	Akap5	0,000000	4,56
ENSMUSG00000032531	Amotl2	0,000000	4,56
ENSMUSG00000027030	Stk39	0,000000	4,54
ENSMUSG00000021176	Efcab11	0,002219	4,53
ENSMUSG00000026109	Tmeff2	0,000000	4,53
ENSMUSG00000042812	Foxf1	0,000035	4,50
ENSMUSG00000026380	Tfcp2l1	0,000023	4,50
ENSMUSG00000026620	Mark1	0,000000	4,49
ENSMUSG00000034883	Lrr1	0,000104	4,44
ENSMUSG00000033327	Tnxb	0,000000	4,43
ENSMUSG00000074457	S100a16	0,000000	4,43
ENSMUSG00000038253	Hoxa5	0,000000	4,43
ENSMUSG00000036223	Ska1	0,000558	4,42
ENSMUSG00000049521	Cdc42ep1	0,000000	4,40
ENSMUSG00000029675	Eln	0,000000	4,40
ENSMUSG00000022231	Sema5a	0,000000	4,39
ENSMUSG00000020182	Ddc	0,000106	4,38
ENSMUSG00000007655	Cav1	0,000000	4,35
ENSMUSG00000027800	Tm4sf1	0,000000	4,33
ENSMUSG00000036856	Wnt4	0,000000	4,31
ENSMUSG00000030074	Gxylt2	0,000000	4,31
ENSMUSG00000002688	Prkd1	0,000002	4,30
ENSMUSG00000042155	Klhl23	0,000023	4,29

ENSMUSG00000021589	Rhobtb3	0,000000	4,29
ENSMUSG00000004951	Hspb1	0,000000	4,27
ENSMUSG00000034684	Sema3f	0,000000	4,25
ENSMUSG00000006457	Actn3	0,000424	4,24
ENSMUSG00000018830	Myh11	0,000224	4,24
ENSMUSG00000035000	Dpp4	0,000549	4,22
ENSMUSG00000001507	Itga3	0,000000	4,21
ENSMUSG00000041064	Pif1	0,000002	4,21
ENSMUSG00000039601	Rcan2	0,000004	4,19
ENSMUSG00000042258	Isl1	0,001613	4,18
ENSMUSG00000027015	Cybrd1	0,000130	4,17
ENSMUSG00000005611	Mrvi1	0,000000	4,17
ENSMUSG00000027827	Kcnab1	0,000000	4,15
ENSMUSG00000058153	Sez6l	0,000001	4,14
ENSMUSG00000027832	Ptx3	0,000000	4,14
ENSMUSG00000039911	Spsb1	0,000000	4,11
ENSMUSG00000025170	Rab40b	0,000355	4,09
ENSMUSG00000046314	Stxbp6	0,000641	4,07
ENSMUSG00000003452	Bicd1	0,000016	4,07
ENSMUSG00000048485	Zbtb8b	0,000000	4,06
ENSMUSG00000037747	Phyhipl	0,000016	4,06
ENSMUSG00000042357	Gjb5	0,000000	4,05
ENSMUSG00000027199	Gatm	0,000000	4,04
ENSMUSG00000043313	Pcdhb19	0,000774	4,04
ENSMUSG00000022033	Pbk	0,000001	4,03
ENSMUSG00000021573	Tppp	0,003225	4,03
ENSMUSG00000042485	Mustn1	0,000050	4,02
ENSMUSG00000048218	Amigo2	0,000000	4,02
ENSMUSG00000037060	Prkcdbp	0,000000	4,00
ENSMUSG00000071656	Lrrn4cl	0,000056	4,00
ENSMUSG00000021186	Fbln5	0,000000	4,00
ENSMUSG00000075254	Heg1	0,000000	3,98
ENSMUSG00000029641	Rasl11a	0,000000	3,98
ENSMUSG00000028780	Sema3c	0,000070	3,98
ENSMUSG00000026235	Epha4	0,000000	3,98
ENSMUSG00000061353	Cxcl12	0,000000	3,98
ENSMUSG00000019929	Dcn	0,000000	3,98
ENSMUSG00000029436	Mmp17	0,000000	3,95
ENSMUSG00000054793	Cadm4	0,000000	3,94
ENSMUSG00000025885	Myo5b	0,002334	3,93
ENSMUSG00000021071	Trim9	0,000019	3,93
ENSMUSG00000021803	Cdhr1	0,000010	3,93
ENSMUSG00000014329	Bicc1	0,000000	3,92
ENSMUSG00000063632	Sox11	0,001667	3,92
ENSMUSG00000042428	Mgat3	0,000000	3,92
ENSMUSG00000096606	Tpbgl	0,000000	3,92
ENSMUSG00000042515	Mum1l1	0,000000	3,91

ENSMUSG00000030790	Adm	0,000002	3,90
ENSMUSG00000019817	Plagl1	0,000113	3,88
ENSMUSG00000020649	Rrm2	0,000000	3,85
ENSMUSG00000046623	Gjb4	0,000227	3,85
ENSMUSG00000049691	Nkx3-2	0,000608	3,83
ENSMUSG00000067377	Tspan6	0,000176	3,82
ENSMUSG00000039405	Prss23	0,000000	3,82
ENSMUSG00000019889	Ptprk	0,000010	3,81
ENSMUSG00000028766	Alpl	0,000000	3,81
ENSMUSG00000024041	Cryaa	0,000000	3,78
ENSMUSG00000028024	Enpep	0,000659	3,77
ENSMUSG00000035305	Ror1	0,000000	3,76
ENSMUSG00000031273	Col4a6	0,000000	3,76
ENSMUSG00000028782	Adgrb2	0,000001	3,75
ENSMUSG00000019124	Scrn1	0,000000	3,75
ENSMUSG00000020627	Klhl29	0,000000	3,73
ENSMUSG00000051043	Gprc5c	0,000238	3,72
ENSMUSG00000030255	Sspn	0,000001	3,72
ENSMUSG00000031561	Tenm3	0,000002	3,72
ENSMUSG00000004631	Sgce	0,000000	3,72
ENSMUSG00000023972	Ptk7	0,000000	3,72
ENSMUSG00000039328	Rnf122	0,000000	3,71
ENSMUSG00000078521	Aunip	0,000068	3,71
ENSMUSG00000001827	Folr1	0,000001	3,71
ENSMUSG00000025964	Adam23	0,000000	3,71
ENSMUSG00000025083	Afap1l2	0,000001	3,70
ENSMUSG00000031990	Jam3	0,000000	3,70
ENSMUSG00000001986	Gria3	0,000000	3,69
ENSMUSG00000016458	Wt1	0,000000	3,68
ENSMUSG00000091243	Vgll3	0,000000	3,68
ENSMUSG00000034903	Cobll1	0,000000	3,68
ENSMUSG00000040875	Osbpl10	0,000056	3,66
ENSMUSG00000031750	Il34	0,000106	3,66
ENSMUSG00000031673	Cdh11	0,000000	3,65
ENSMUSG00000005338	Cadm3	0,000000	3,65
ENSMUSG00000028664	Ephb2	0,000000	3,65
ENSMUSG00000046807	Lrrc75b	0,000391	3,65
ENSMUSG00000015854	Cd5l	0,000000	3,64
ENSMUSG00000034910	Pygo1	0,000314	3,63
ENSMUSG00000038463	Olfml2b	0,000000	3,63
ENSMUSG00000031595	Pdgfrl	0,000006	3,63
ENSMUSG00000029287	Tgfbr3	0,000000	3,63
ENSMUSG00000067586	S1pr3	0,000000	3,63
ENSMUSG00000070576	Mn1	0,001108	3,62
ENSMUSG00000049907	Rasl11b	0,000053	3,62
ENSMUSG00000031434	Morc4	0,000010	3,61
ENSMUSG00000033389	Arhgap44	0,000030	3,60

ENSMUSG00000006931	P3h4	0,000000	3,59
ENSMUSG00000022425	Enpp2	0,000000	3,59
ENSMUSG00000070802	Pnmal2	0,000000	3,58
ENSMUSG00000031099	Smarca1	0,002726	3,58
ENSMUSG00000029005	Draxin	0,000037	3,57
ENSMUSG00000022261	Sdc2	0,000000	3,56
ENSMUSG00000044550	Tceal3	0,000138	3,54
ENSMUSG00000020241	Col6a2	0,000000	3,53
ENSMUSG00000024427	Spry4	0,000003	3,53
ENSMUSG00000068876	Cgn	0,000000	3,53
ENSMUSG00000045246	Kcng4	0,002091	3,53
ENSMUSG00000021798	Ldb3	0,004218	3,52
ENSMUSG00000032327	Stra6	0,000001	3,52
ENSMUSG00000062309	Rpp25	0,000010	3,51
ENSMUSG00000044676	Zfp612	0,000759	3,51
ENSMUSG00000034839	Larp6	0,004457	3,50
ENSMUSG00000079560	Hoxa3	0,000099	3,50
ENSMUSG00000020185	E2f7	0,000071	3,50
ENSMUSG00000027859	Ngf	0,000515	3,49
ENSMUSG00000041559	Fmod	0,000000	3,49
ENSMUSG00000032011	Thy1	0,000000	3,48
ENSMUSG00000029581	Fscn1	0,000000	3,48
ENSMUSG00000045790	Ccdc149	0,000104	3,48
ENSMUSG00000026399	Cd55	0,000000	3,47
ENSMUSG00000022203	Efs	0,000435	3,47
ENSMUSG00000033585	Ndn	0,000000	3,45
ENSMUSG00000060227	Casc4	0,000000	3,45
ENSMUSG00000020176	Grb10	0,000000	3,44
ENSMUSG00000060961	Slc4a4	0,000130	3,44
ENSMUSG00000039835	Nhsl1	0,000001	3,44
ENSMUSG00000054252	Fgfr3	0,001925	3,42
ENSMUSG00000051000	Fam160a1	0,000663	3,41
ENSMUSG00000005958	Ephb3	0,000001	3,41
ENSMUSG00000022034	Esco2	0,000109	3,41
ENSMUSG00000003070	Efna2	0,000103	3,40
ENSMUSG00000032271	Nnmt	0,000000	3,40
ENSMUSG00000038349	Plcl1	0,000064	3,39
ENSMUSG00000046470	Sox18	0,000001	3,39
ENSMUSG00000030772	Dkk3	0,000000	3,39
ENSMUSG00000071984	Fndc1	0,000000	3,38
ENSMUSG00000028883	Sema3a	0,000000	3,38
ENSMUSG00000066842	Hmcn1	0,001142	3,37
ENSMUSG00000039831	Arhgap29	0,000000	3,36
ENSMUSG00000033295	Ptprf	0,000000	3,36
ENSMUSG00000026185	Igfbp5	0,000894	3,36
ENSMUSG00000018387	Shroom1	0,001936	3,36
ENSMUSG00000033965	Slc16a2	0,000000	3,36

ENSMUSG00000015652	Steap1	0,000000	3,36
ENSMUSG00000027546	Atp9a	0,000000	3,35
ENSMUSG00000039607	Rbms3	0,000000	3,35
ENSMUSG00000061048	Cdh3	0,000000	3,35
ENSMUSG00000033578	Tmem35a	0,000032	3,35
ENSMUSG00000021994	Wnt5a	0,000001	3,35
ENSMUSG00000030247	Kcnj8	0,000635	3,35
ENSMUSG00000024500	Ppp2r2b	0,000388	3,35
ENSMUSG00000003534	Ddr1	0,000000	3,35
ENSMUSG00000035783	Acta2	0,000000	3,34
ENSMUSG00000018593	Sparc	0,000000	3,34
ENSMUSG00000054855	Rnd1	0,000000	3,34
ENSMUSG00000036158	Prickle1	0,000000	3,33
ENSMUSG00000005836	Gata6	0,000000	3,33
ENSMUSG00000035873	Pawr	0,000002	3,33
ENSMUSG00000038235	F11r	0,000000	3,33
ENSMUSG00000043635	Adamts3	0,001884	3,33
ENSMUSG00000032092	Mpzl2	0,000011	3,32
ENSMUSG00000029718	Pcolce	0,000000	3,32
ENSMUSG00000034586	Hid1	0,001412	3,31
ENSMUSG00000031119	Gpc4	0,000001	3,31
ENSMUSG00000030022	Adamts9	0,000000	3,31
ENSMUSG00000054728	Phactr1	0,001770	3,31
ENSMUSG00000074364	Ehd2	0,000000	3,30
ENSMUSG00000027306	Nusap1	0,000000	3,30
ENSMUSG00000001119	Col6a1	0,000000	3,30
ENSMUSG00000027408	Cpxm1	0,000000	3,30
ENSMUSG00000035373	Ccl7	0,000000	3,30
ENSMUSG00000070803	Cited4	0,000006	3,29
ENSMUSG00000002808	Epdr1	0,000003	3,29
ENSMUSG00000061451	Tmem151a	0,000000	3,29
ENSMUSG00000020808	Fam64a	0,000000	3,29
ENSMUSG00000023092	Fhl1	0,000669	3,28
ENSMUSG00000030643	Rab30	0,000043	3,28
ENSMUSG00000032717	Mdfi	0,000005	3,28
ENSMUSG00000032816	Igdcc4	0,000000	3,28
ENSMUSG00000074476	Spc24	0,000005	3,27
ENSMUSG00000001435	Col18a1	0,000000	3,27
ENSMUSG00000038236	Hoxa7	0,000315	3,26
ENSMUSG00000017493	Igfbp4	0,000000	3,26
ENSMUSG00000042834	Nrep	0,000222	3,26
ENSMUSG00000000942	Hoxa4	0,000730	3,25
ENSMUSG00000029765	Plxna4	0,000000	3,25
ENSMUSG00000038521	C1s1	0,000000	3,25
ENSMUSG00000022297	Fzd6	0,000317	3,25
ENSMUSG00000010830	Kdelr3	0,000000	3,25
ENSMUSG00000041548	Hspb8	0,000000	3,25

ENSMUSG00000006435	Neurl1a	0,000566	3,24
ENSMUSG00000037706	Cd81	0,000000	3,24
ENSMUSG00000028860	Sytl1	0,000517	3,23
ENSMUSG00000017446	C1qtnf1	0,000000	3,23
ENSMUSG00000057614	Gnai1	0,000434	3,22
ENSMUSG00000028633	Ctps	0,000000	3,22
ENSMUSG00000031112	Stk26	0,000000	3,21
ENSMUSG00000025207	Sema4g	0,000679	3,21
ENSMUSG00000001403	Ube2c	0,000000	3,21
ENSMUSG00000043531	Sorcs1	0,000085	3,21
ENSMUSG00000028033	Kcnq5	0,002813	3,20
ENSMUSG00000036106	Prr5	0,000093	3,20
ENSMUSG00000039103	Nexn	0,000551	3,20
ENSMUSG00000001020	S100a4	0,000003	3,20
ENSMUSG00000028399	Ptprd	0,000004	3,19
ENSMUSG00000037379	Spon2	0,002121	3,19
ENSMUSG00000031274	Col4a5	0,000000	3,18
ENSMUSG00000030352	Tspan9	0,000000	3,18
ENSMUSG00000029710	Ephb4	0,000000	3,18
ENSMUSG00000046532	Ar	0,000794	3,18
ENSMUSG00000024087	Cyp1b1	0,000000	3,18
ENSMUSG00000050288	Fzd2	0,000000	3,18
ENSMUSG00000054675	Tmem119	0,000000	3,17
ENSMUSG00000046818	Ddit4l	0,000133	3,17
ENSMUSG00000017716	Birc5	0,000000	3,17
ENSMUSG00000034486	Gbx2	0,000218	3,17
ENSMUSG00000028175	Depdc1a	0,000158	3,17
ENSMUSG00000007872	Id3	0,000000	3,16
ENSMUSG00000032372	Plscr2	0,000000	3,16
ENSMUSG00000024538	Ppic	0,000000	3,16
ENSMUSG00000034573	Ptpn13	0,000000	3,15
ENSMUSG00000068699	Flnc	0,000000	3,15
ENSMUSG00000045629	Sh3tc2	0,000036	3,15
ENSMUSG00000028195	Cyr61	0,000000	3,15
ENSMUSG00000031070	Mrgprf	0,000000	3,15
ENSMUSG00000055044	Pdlim1	0,000000	3,14
ENSMUSG00000041329	Atp1b2	0,000561	3,14
ENSMUSG00000073802	Cdkn2b	0,000001	3,13
ENSMUSG00000032625	Thsd7a	0,000180	3,13
ENSMUSG00000019942	Cdk1	0,000000	3,13
ENSMUSG00000006651	Aplp1	0,000000	3,13
ENSMUSG00000072980	Oip5	0,000444	3,12
ENSMUSG00000044303	Cdkn2a	0,000005	3,12
ENSMUSG00000036902	Neto2	0,000029	3,11
ENSMUSG00000049598	Vsig8	0,000027	3,11
ENSMUSG00000074676	Foxs1	0,000000	3,09
ENSMUSG00000029659	Medag	0,001216	3,09

ENSMUSG00000019539	Rcn3	0,000000	3,09
ENSMUSG00000048376	F2r	0,000000	3,09
ENSMUSG00000021047	Nova1	0,003576	3,08
ENSMUSG00000071176	Arhgef10	0,000005	3,08
ENSMUSG00000051817	Sox12	0,000000	3,08
ENSMUSG00000021944	Gata4	0,002011	3,07
ENSMUSG00000019831	Wasf1	0,000010	3,07
ENSMUSG00000006369	Fbln1	0,000004	3,07
ENSMUSG00000041540	Sox5	0,003003	3,07
ENSMUSG00000022324	Matn2	0,000406	3,06
ENSMUSG00000072964	Bhlhb9	0,000006	3,06
ENSMUSG00000030770	Parva	0,000000	3,06
ENSMUSG00000036777	Anln	0,000000	3,05
ENSMUSG00000022479	Vdr	0,000024	3,04
ENSMUSG00000021760	Gpx8	0,000000	3,04
ENSMUSG00000016262	Sertad4	0,000383	3,04
ENSMUSG00000027210	Meis2	0,000004	3,04
ENSMUSG00000031298	Adgrg2	0,003712	3,03
ENSMUSG00000027316	Gfra4	0,001966	3,02
ENSMUSG00000020493	Prr11	0,000000	3,02
ENSMUSG00000069171	Nr2f1	0,001846	3,02
ENSMUSG00000021697	Depdc1b	0,001586	3,02
ENSMUSG00000057880	Abat	0,000010	3,02
ENSMUSG00000022306	Zfpm2	0,000002	3,01
ENSMUSG00000022836	Mylk	0,000016	3,00
ENSMUSG00000047986	Palm3	0,001192	3,00
ENSMUSG00000032783	Troap	0,000080	3,00
ENSMUSG00000022505	Emp2	0,000000	3,00
ENSMUSG00000028262	Clca3a2	0,000004	3,00
ENSMUSG00000047907	Tshz2	0,000000	2,99
ENSMUSG00000025140	Pycr1	0,000007	2,99
ENSMUSG00000025738	Fbxl16	0,000253	2,99
ENSMUSG00000039518	Cdsn	0,002204	2,98
ENSMUSG00000017144	Rnd3	0,000000	2,98
ENSMUSG00000021965	Ska3	0,000065	2,98
ENSMUSG00000032547	Ryk	0,000000	2,98
ENSMUSG00000020330	Hmmr	0,000000	2,98
ENSMUSG00000025272	Tro	0,001414	2,97
ENSMUSG00000008575	Nfib	0,000000	2,97
ENSMUSG00000028832	Stmn1	0,000000	2,97
ENSMUSG00000038119	Cdon	0,000000	2,96
ENSMUSG00000028128	F3	0,000001	2,96
ENSMUSG00000052539	Magi3	0,000000	2,96
ENSMUSG00000032702	Kank1	0,000027	2,96
ENSMUSG00000057969	Sema3b	0,004236	2,96
ENSMUSG00000046387	Pcdhb17	0,001483	2,95
ENSMUSG00000027840	Wnt2b	0,002377	2,95

ENSMUSG00000031284	Pak3	0,003857	2,95
ENSMUSG00000042942	Greb1l	0,004542	2,94
ENSMUSG00000026443	Lrrn2	0,000000	2,94
ENSMUSG00000015653	Steap2	0,000000	2,94
ENSMUSG00000038379	Ttk	0,001045	2,94
ENSMUSG00000032243	Itga11	0,002629	2,93
ENSMUSG00000051236	Msrb3	0,000001	2,93
ENSMUSG00000079343	C1s2	0,000001	2,93
ENSMUSG00000033855	Ston1	0,000001	2,93
ENSMUSG00000001761	Smo	0,000000	2,92
ENSMUSG00000023960	Enpp5	0,000000	2,92
ENSMUSG00000026042	Col5a2	0,000000	2,91
ENSMUSG00000038943	Prc1	0,000000	2,91
ENSMUSG00000032218	Ccnb2	0,000000	2,91
ENSMUSG00000031520	Vegfc	0,000000	2,91
ENSMUSG00000029122	Evc	0,000030	2,90
ENSMUSG00000020123	Avpr1a	0,000770	2,90
ENSMUSG00000078452	Raet1d	0,000183	2,90
ENSMUSG00000021913	Ogdhl	0,001277	2,90
ENSMUSG00000045273	Cenph	0,000981	2,90
ENSMUSG00000014932	Yes1	0,000868	2,89
ENSMUSG00000025887	Casp12	0,000013	2,89
ENSMUSG00000055320	Tead1	0,000000	2,89
ENSMUSG00000053219	Raet1e	0,000135	2,89
ENSMUSG00000037712	Fermt2	0,000000	2,88
ENSMUSG00000001604	Tcea3	0,000322	2,88
ENSMUSG00000002249	Tead3	0,000002	2,88
ENSMUSG00000026343	Gpr39	0,000000	2,87
ENSMUSG00000035365	Parpbp	0,000172	2,87
ENSMUSG00000026344	Lypd1	0,000000	2,87
ENSMUSG00000037010	Apln	0,002382	2,86
ENSMUSG00000038074	Fkbp14	0,000000	2,86
ENSMUSG00000056427	Slit3	0,000000	2,86
ENSMUSG00000030409	Dmpk	0,000000	2,86
ENSMUSG00000023367	Tmem176a	0,000000	2,85
ENSMUSG00000006445	Epha2	0,000000	2,85
ENSMUSG00000002006	Pdzd4	0,002345	2,85
ENSMUSG00000031239	Itm2a	0,000289	2,84
ENSMUSG00000035683	Melk	0,000016	2,84
ENSMUSG00000050910	Cdr2l	0,000000	2,84
ENSMUSG00000032334	Loxl1	0,000000	2,83
ENSMUSG00000012443	Kif11	0,000000	2,83
ENSMUSG00000022793	B4galt4	0,000000	2,83
ENSMUSG00000064264	Zfp428	0,000095	2,83
ENSMUSG00000032289	Thsd4	0,000692	2,82
ENSMUSG00000070436	Serpinh1	0,000000	2,82
ENSMUSG00000078670	Fam174b	0,000589	2,82

ENSMUSG00000043099	Hic1	0,000000	2,81
ENSMUSG00000028601	Echdc2	0,000792	2,81
ENSMUSG00000001555	Fkbp10	0,000000	2,81
ENSMUSG00000027803	Wwtr1	0,000001	2,81
ENSMUSG00000055799	Tcf7l1	0,000026	2,81
ENSMUSG00000039252	Lgi2	0,000001	2,81
ENSMUSG00000035799	Twist1	0,000024	2,80
ENSMUSG00000059602	Syn3	0,000000	2,80
ENSMUSG00000033436	Armcx2	0,000000	2,80
ENSMUSG00000042745	Id1	0,000000	2,80
ENSMUSG00000044927	H1fx	0,000076	2,80
ENSMUSG00000034773	BC030867	0,000495	2,80
ENSMUSG00000032216	Nedd4	0,000000	2,80
ENSMUSG00000040447	Spns2	0,000039	2,79
ENSMUSG00000070509	Rgma	0,000108	2,79
ENSMUSG00000018648	Dusp14	0,001276	2,79
ENSMUSG00000032085	Tagln	0,000000	2,79
ENSMUSG00000035455	Fignl1	0,000000	2,79
ENSMUSG00000034520	Gjc1	0,000180	2,79
ENSMUSG00000024304	Cdh2	0,000000	2,78
ENSMUSG00000073599	Ecscr	0,000025	2,78
ENSMUSG00000052062	Pard3b	0,003708	2,77
ENSMUSG00000045725	Prr15	0,001348	2,77
ENSMUSG00000017969	Ptgis	0,000000	2,77
ENSMUSG00000020914	Top2a	0,000000	2,77
ENSMUSG00000050370	Ch25h	0,000005	2,77
ENSMUSG00000038793	Lefty1	0,000033	2,76
ENSMUSG00000036098	Myrf	0,000000	2,76
ENSMUSG00000027805	Pfn2	0,000000	2,76
ENSMUSG00000020439	Smtn	0,000000	2,76
ENSMUSG00000036768	Kif15	0,000281	2,75
ENSMUSG00000027661	Slc2a10	0,000648	2,75
ENSMUSG00000053931	Cnn3	0,000000	2,75
ENSMUSG00000015217	Hmgb3	0,000063	2,75
ENSMUSG00000037784	Dzip1l	0,000197	2,75
ENSMUSG00000022178	Ajuba	0,000003	2,75
ENSMUSG00000061740	Cyp2d22	0,001277	2,74
ENSMUSG00000026589	Sec16b	0,002048	2,74
ENSMUSG00000028369	Svep1	0,000379	2,74
ENSMUSG00000000690	Hoxb6	0,000995	2,74
ENSMUSG00000028807	Zbtb8a	0,000000	2,74
ENSMUSG00000029381	Shroom3	0,002280	2,73
ENSMUSG00000044548	Dact1	0,001051	2,73
ENSMUSG00000032549	Rab6b	0,004946	2,72
ENSMUSG00000020814	Mxra7	0,000472	2,72
ENSMUSG00000056481	Cd248	0,000000	2,72
ENSMUSG00000029061	Mmp23	0,000000	2,72

ENSMUSG00000021253	Tgfb3	0,000000	2,71
ENSMUSG00000028476	Reck	0,000205	2,71
ENSMUSG00000067818	Myl9	0,000000	2,71
ENSMUSG00000031209	Heph	0,000684	2,71
ENSMUSG00000034311	Kif4	0,000057	2,71
ENSMUSG00000078794	Dact3	0,000016	2,70
ENSMUSG00000032401	Lctl	0,000993	2,70
ENSMUSG00000023919	Cenpq	0,000570	2,70
ENSMUSG00000028073	Pear1	0,000001	2,69
ENSMUSG00000046295	Ankle1	0,001107	2,69
ENSMUSG00000020937	Plcd3	0,004237	2,69
ENSMUSG00000006398	Cdc20	0,000001	2,69
ENSMUSG00000031523	Dlc1	0,000008	2,69
ENSMUSG00000026779	Mastl	0,000178	2,68
ENSMUSG00000029126	Nsg1	0,000008	2,68
ENSMUSG00000062312	Erbb2	0,000030	2,68
ENSMUSG00000030867	Plk1	0,000001	2,68
ENSMUSG00000029231	Pdgfra	0,000000	2,67
ENSMUSG00000034206	Polq	0,000430	2,66
ENSMUSG00000003352	Cacnb3	0,000000	2,66
ENSMUSG00000042842	Serpinb6b	0,000000	2,66
ENSMUSG00000034023	Fancd2	0,002096	2,66
ENSMUSG00000031217	Efnb1	0,000000	2,66
ENSMUSG00000047557	Lxn	0,000000	2,66
ENSMUSG00000020160	Meis1	0,000005	2,65
ENSMUSG00000075394	Hoxc4	0,004483	2,65
ENSMUSG00000052911	Lamb2	0,000000	2,65
ENSMUSG00000061751	Kalrn	0,000161	2,65
ENSMUSG00000003355	Fkbp11	0,000144	2,65
ENSMUSG00000020844	Nxn	0,000000	2,64
ENSMUSG00000024590	Lmnb1	0,000001	2,64
ENSMUSG00000029380	Cxcl1	0,000000	2,64
ENSMUSG00000027379	Bub1	0,000199	2,63
ENSMUSG00000023940	Sgol1	0,000622	2,63
ENSMUSG00000027699	Ect2	0,000190	2,63
ENSMUSG00000027654	Fam83d	0,000084	2,63
ENSMUSG00000023505	Cdca3	0,000001	2,63
ENSMUSG00000074743	Thbd	0,000000	2,63
ENSMUSG00000072082	Ccnf	0,000000	2,63
ENSMUSG00000022687	Boc	0,000232	2,62
ENSMUSG00000034485	Uaca	0,000006	2,62
ENSMUSG00000020122	Egfr	0,000000	2,62
ENSMUSG00000020186	Csrp2	0,000001	2,62
ENSMUSG00000015709	Arnt2	0,000284	2,62
ENSMUSG00000042284	Itga1	0,000089	2,62
ENSMUSG00000048327	Ckap2l	0,000003	2,62
ENSMUSG00000037725	Ckap2	0,000033	2,61

ENSMUSG00000020695	Mrc2	0,000000	2,61
ENSMUSG00000029810	Tmem176b	0,000000	2,61
ENSMUSG00000002055	Spag5	0,000263	2,61
ENSMUSG00000031861	Lpar2	0,000809	2,61
ENSMUSG00000026674	Ddr2	0,000000	2,61
ENSMUSG00000024521	Pmaip1	0,000000	2,61
ENSMUSG00000029769	Ccdc136	0,003712	2,60
ENSMUSG00000033453	Adamts15	0,000065	2,60
ENSMUSG00000027220	Syt13	0,000000	2,60
ENSMUSG00000041117	Ccdc8	0,000110	2,59
ENSMUSG00000024056	Ndc80	0,000314	2,59
ENSMUSG00000020044	Timp3	0,000001	2,59
ENSMUSG00000018378	Cuedc1	0,000047	2,59
ENSMUSG00000029859	Epha1	0,000112	2,59
ENSMUSG00000022322	Shcbp1	0,000876	2,58
ENSMUSG00000044646	Zbtb7c	0,000001	2,58
ENSMUSG00000031955	Bcar1	0,000001	2,58
ENSMUSG00000051279	Gdf6	0,000595	2,58
ENSMUSG00000024989	Cep55	0,000018	2,58
ENSMUSG00000027860	Vangl1	0,000323	2,58
ENSMUSG00000053062	Jam2	0,003091	2,58
ENSMUSG00000039476	Prrx2	0,000387	2,58
ENSMUSG00000013921	Clip3	0,000006	2,58
ENSMUSG00000029832	Nfe2l3	0,004958	2,57
ENSMUSG00000030246	Ldhb	0,000000	2,57
ENSMUSG00000031877	Ces2g	0,000034	2,57
ENSMUSG00000028194	Ddah1	0,000392	2,56
ENSMUSG00000026669	Mcm10	0,000329	2,56
ENSMUSG00000036867	Smad6	0,000007	2,56
ENSMUSG00000036036	Zfp57	0,000450	2,56
ENSMUSG00000024665	Fads2	0,000001	2,56
ENSMUSG00000033149	Phldb2	0,000000	2,55
ENSMUSG00000022021	Diaph3	0,000043	2,55
ENSMUSG00000026981	Il1rn	0,000000	2,55
ENSMUSG00000021796	Bmpr1a	0,000000	2,54
ENSMUSG00000036459	Wtip	0,000027	2,54
ENSMUSG00000026622	Nek2	0,000027	2,54
ENSMUSG00000022146	Osmr	0,000001	2,54
ENSMUSG00000046768	Rhoj	0,000000	2,54
ENSMUSG00000062300	Nectin2	0,000000	2,54
ENSMUSG00000032254	Kif23	0,000026	2,53
ENSMUSG00000036256	Igfbp7	0,000000	2,53
ENSMUSG00000029070	Mxra8	0,000000	2,53
ENSMUSG00000036995	Asap3	0,000041	2,53
ENSMUSG00000041741	Pde3a	0,001516	2,53
ENSMUSG00000034765	Dusp5	0,000008	2,53
ENSMUSG00000044033	Ccdc141	0,001271	2,53

ENSMUSG00000025213	Kazald1	0,001678	2,53
ENSMUSG00000013419	Zfp651	0,000072	2,52
ENSMUSG00000032060	Cryab	0,000000	2,51
ENSMUSG00000051220	Ercc6l	0,000026	2,51
ENSMUSG00000032340	Neo1	0,000000	2,51
ENSMUSG00000037474	Dtl	0,000386	2,51
ENSMUSG00000000753	Serpinf1	0,000000	2,50
ENSMUSG00000038587	Akap12	0,000421	2,50
ENSMUSG00000032725	Folr2	0,000021	2,50
ENSMUSG00000027002	Nckap1	0,000006	2,49
ENSMUSG00000069835	Sat2	0,004237	2,49
ENSMUSG00000051375	Pcdh1	0,000055	2,49
ENSMUSG00000026576	Atp1b1	0,000004	2,49
ENSMUSG00000005233	Spc25	0,000007	2,49
ENSMUSG00000027496	Aurka	0,000001	2,49
ENSMUSG00000026683	Nuf2	0,000057	2,49
ENSMUSG00000021765	Fst	0,001993	2,49
ENSMUSG00000028464	Tpm2	0,000001	2,48
ENSMUSG00000041889	Shisa4	0,000008	2,48
ENSMUSG00000034463	Scara3	0,000018	2,48
ENSMUSG00000025747	Tyms	0,000025	2,47
ENSMUSG00000026208	Des	0,000000	2,47
ENSMUSG00000023015	Racgap1	0,000000	2,47
ENSMUSG00000028402	Mpdz	0,000784	2,47
ENSMUSG00000036502	Tmem255a	0,001825	2,47
ENSMUSG00000055737	Ghr	0,001383	2,46
ENSMUSG00000068115	Ninl	0,000027	2,46
ENSMUSG00000038776	Ephx1	0,000000	2,46
ENSMUSG00000048285	Frmd6	0,000000	2,46
ENSMUSG00000034457	Eda2r	0,000842	2,46
ENSMUSG00000027313	Chac1	0,000478	2,46
ENSMUSG00000024177	Nme4	0,000015	2,45
ENSMUSG00000029153	Ociad2	0,004391	2,45
ENSMUSG00000030978	Rrm1	0,000000	2,45
ENSMUSG00000017009	Sdc4	0,000000	2,45
ENSMUSG00000028678	Kif2c	0,000156	2,45
ENSMUSG00000050315	Synpo2	0,003985	2,45
ENSMUSG00000006403	Adamts4	0,000278	2,45
ENSMUSG00000046402	Rbp1	0,000001	2,45
ENSMUSG00000016349	Eef1a2	0,001558	2,44
ENSMUSG00000047963	Stbd1	0,000016	2,44
ENSMUSG00000038668	Lpar1	0,000280	2,44
ENSMUSG00000040488	Ltbp4	0,000605	2,44
ENSMUSG00000037003	Tns2	0,000057	2,43
ENSMUSG00000032714	Syde1	0,000006	2,43
ENSMUSG00000070348	Ccnd1	0,000010	2,43
ENSMUSG00000041498	Kif14	0,004388	2,43

ENSMUSG00000047238	Mageh1	0,000143	2,43
ENSMUSG00000000325	Arvcf	0,000000	2,43
ENSMUSG00000028044	Cks1b	0,000000	2,43
ENSMUSG00000019779	Frk	0,001770	2,42
ENSMUSG00000019846	Lama4	0,000013	2,42
ENSMUSG00000017667	Zfp334	0,000463	2,41
ENSMUSG00000025555	Farp1	0,000014	2,41
ENSMUSG00000027962	Vcam1	0,000003	2,41
ENSMUSG00000021745	Ptprg	0,000290	2,41
ENSMUSG00000031642	Sh3rf1	0,000008	2,41
ENSMUSG00000089715	Cbx6	0,000000	2,41
ENSMUSG00000038020	Rapgefl1	0,003664	2,41
ENSMUSG00000071856	Mcc	0,000657	2,41
ENSMUSG00000001228	Uhrf1	0,000001	2,41
ENSMUSG00000006356	Crip2	0,000047	2,40
ENSMUSG00000044734	Serpinb1a	0,001884	2,40
ENSMUSG00000027200	Sema6d	0,000164	2,40
ENSMUSG00000037225	Fgf2	0,000005	2,39
ENSMUSG00000024253	Dync2li1	0,002001	2,39
ENSMUSG00000007039	Ddah2	0,000010	2,39
ENSMUSG00000031342	Gpm6b	0,004724	2,39
ENSMUSG00000037032	Apbb1	0,000484	2,39
ENSMUSG00000015647	Lama5	0,000896	2,38
ENSMUSG00000035441	Myo1d	0,000046	2,38
ENSMUSG00000001300	Efnb2	0,000006	2,38
ENSMUSG00000031078	Cttn	0,000000	2,38
ENSMUSG00000034906	Ncaph	0,000180	2,37
ENSMUSG00000037621	Atoh8	0,000027	2,37
ENSMUSG00000072949	Acot1	0,000176	2,37
ENSMUSG00000020492	Ska2	0,000036	2,37
ENSMUSG00000041842	Fhdc1	0,000030	2,36
ENSMUSG00000053198	Prx	0,004414	2,36
ENSMUSG00000021464	Ror2	0,000141	2,36
ENSMUSG00000030641	Ddias	0,002554	2,36
ENSMUSG00000048922	Cdca2	0,000251	2,35
ENSMUSG00000035385	Ccl2	0,000003	2,35
ENSMUSG00000027331	Knstrn	0,000027	2,35
ENSMUSG00000024620	Pdgfrb	0,000001	2,35
ENSMUSG00000023224	Serping1	0,000000	2,35
ENSMUSG00000021318	Gli3	0,001412	2,35
ENSMUSG00000026494	Kif26b	0,000292	2,34
ENSMUSG00000017314	Mpp2	0,000504	2,34
ENSMUSG00000043671	Dpy19l3	0,000371	2,34
ENSMUSG00000051378	Kif18b	0,002677	2,33
ENSMUSG00000025812	Pard3	0,001987	2,33
ENSMUSG00000042734	Ttc9	0,000421	2,33
ENSMUSG00000002900	Lamb1	0,000010	2,33

ENSMUSG00000062248	Cks2	0,000180	2,32
ENSMUSG00000029752	Asns	0,000000	2,32
ENSMUSG00000020205	Phlda1	0,000033	2,32
ENSMUSG00000000253	Gmpr	0,000751	2,32
ENSMUSG00000028197	Col24a1	0,000051	2,32
ENSMUSG00000015094	Npdc1	0,000006	2,32
ENSMUSG00000031024	St5	0,000011	2,31
ENSMUSG00000019467	Arhgef25	0,000072	2,31
ENSMUSG00000019718	L3hypdh	0,001259	2,31
ENSMUSG00000027223	Mapk8ip1	0,000016	2,31
ENSMUSG00000033420	Antxr1	0,000026	2,31
ENSMUSG00000024664	Fads3	0,000006	2,30
ENSMUSG00000031756	Cenpn	0,002604	2,30
ENSMUSG00000039646	Vasn	0,000001	2,30
ENSMUSG00000059791	Nrm	0,000045	2,29
ENSMUSG00000031562	Dctd	0,003700	2,29
ENSMUSG00000032875	Arhgef17	0,000021	2,29
ENSMUSG00000038677	Scube3	0,000396	2,29
ENSMUSG00000022074	Tnfrsf10b	0,000271	2,29
ENSMUSG00000020674	Pxdn	0,000015	2,28
ENSMUSG00000055612	Cdca7	0,000122	2,28
ENSMUSG00000029761	Cald1	0,000008	2,28
ENSMUSG00000042489	Clspn	0,000872	2,27
ENSMUSG00000026196	Bard1	0,004939	2,27
ENSMUSG00000053646	Plxnb1	0,000659	2,27
ENSMUSG00000050953	Gja1	0,000031	2,27
ENSMUSG00000058070	Eml1	0,000119	2,27
ENSMUSG00000005470	Asf1b	0,000016	2,27
ENSMUSG00000041431	Ccnb1	0,000009	2,26
ENSMUSG00000002732	Fkbp7	0,000521	2,26
ENSMUSG00000032586	Traip	0,004082	2,26
ENSMUSG00000009614	Sardh	0,000056	2,26
ENSMUSG00000027469	Tpx2	0,000001	2,26
ENSMUSG00000041444	Arhgap32	0,002011	2,26
ENSMUSG00000059901	Adamts14	0,000106	2,26
ENSMUSG00000054263	Lifr	0,000970	2,26
ENSMUSG00000047749	Zc3hav1l	0,003745	2,25
ENSMUSG00000024222	Fkbp5	0,000000	2,25
ENSMUSG00000032374	Plod2	0,000004	2,25
ENSMUSG00000027221	Chst1	0,000307	2,25
ENSMUSG00000060600	Eno3	0,000011	2,25
ENSMUSG00000022440	C1qtnf6	0,000001	2,25
ENSMUSG00000050410	Tcf19	0,000086	2,25
ENSMUSG00000030281	Il17rc	0,003133	2,25
ENSMUSG00000014599	Csf1	0,000002	2,24
ENSMUSG00000042215	Bag2	0,000145	2,24
ENSMUSG00000060935	Tmem263	0,000001	2,24

ENSMUSG00000042363	Lgalsl	0,003209	2,24
ENSMUSG00000020105	Lrig3	0,004154	2,23
ENSMUSG00000034317	Trim59	0,000074	2,23
ENSMUSG00000035274	Tpbg	0,000814	2,23
ENSMUSG00000028551	Cdkn2c	0,000000	2,22
ENSMUSG00000019997	Ctgf	0,000035	2,22
ENSMUSG00000089762	Ier5l	0,000029	2,22
ENSMUSG00000050530	Fam171a1	0,000028	2,22
ENSMUSG00000049577	Zfpm1	0,000061	2,22
ENSMUSG00000048644	Ctxn1	0,000364	2,22
ENSMUSG00000029661	Col1a2	0,000000	2,21
ENSMUSG00000030208	Emp1	0,000000	2,21
ENSMUSG00000017176	Nt5c3b	0,000002	2,21
ENSMUSG00000018672	Copz2	0,000193	2,21
ENSMUSG00000038418	Egr1	0,000002	2,21
ENSMUSG00000053110	Yap1	0,000030	2,21
ENSMUSG00000021707	Dhfr	0,000491	2,21
ENSMUSG00000022994	Adcy6	0,000040	2,21
ENSMUSG00000027203	Dut	0,000168	2,21
ENSMUSG00000033209	Ttc28	0,000573	2,20
ENSMUSG00000002980	Bcam	0,000073	2,20
ENSMUSG00000026211	Obsl1	0,000191	2,20
ENSMUSG00000004665	Cnn2	0,000000	2,19
ENSMUSG00000026072	Il1r1	0,000003	2,19
ENSMUSG00000034205	Loxl2	0,000004	2,19
ENSMUSG00000024640	Psat1	0,000001	2,19
ENSMUSG00000001025	S100a6	0,000000	2,19
ENSMUSG00000044026	Slc35g1	0,001963	2,19
ENSMUSG00000018861	Fdxr	0,000004	2,19
ENSMUSG00000027074	Slc43a3	0,000040	2,19
ENSMUSG00000055723	Rras2	0,000005	2,19
ENSMUSG00000026605	Cenpf	0,000327	2,18
ENSMUSG00000074480	Mex3a	0,000917	2,17
ENSMUSG00000031375	Bgn	0,000000	2,17
ENSMUSG00000020834	Dhrs13	0,002761	2,17
ENSMUSG00000022594	Lynx1	0,000364	2,17
ENSMUSG00000031262	Cenpi	0,003374	2,16
ENSMUSG00000013089	Etv5	0,000018	2,15
ENSMUSG00000053398	Phgdh	0,000005	2,15
ENSMUSG00000033952	Aspm	0,001934	2,15
ENSMUSG00000033565	Rbfox2	0,000030	2,15
ENSMUSG00000027993	Trim2	0,000164	2,15
ENSMUSG00000020473	Aebp1	0,000019	2,15
ENSMUSG00000024043	Arhgap28	0,001432	2,15
ENSMUSG00000027715	Ccna2	0,000000	2,15
ENSMUSG00000046329	Slc25a23	0,000150	2,15
ENSMUSG00000026604	Ptpn14	0,000158	2,15

ENSMUSG00000040118	Cacna2d1	0,003438	2,14
ENSMUSG00000026873	Phf19	0,002127	2,14
ENSMUSG00000019853	Hebp2	0,004580	2,14
ENSMUSG00000054717	Hmgb2	0,000000	2,14
ENSMUSG00000032411	Tfdp2	0,000109	2,13
ENSMUSG00000074682	Zcchc3	0,000686	2,13
ENSMUSG00000013584	Aldh1a2	0,000439	2,12
ENSMUSG00000037544	Dlgap5	0,000536	2,12
ENSMUSG00000021838	Samd4	0,003914	2,12
ENSMUSG00000030029	Lrig1	0,000973	2,12
ENSMUSG00000028218	Fam92a	0,002709	2,11
ENSMUSG00000032400	Zwilch	0,000862	2,11
ENSMUSG00000001517	Foxm1	0,000059	2,11
ENSMUSG00000041845	Rhod	0,001348	2,11
ENSMUSG00000040690	Col16a1	0,001255	2,11
ENSMUSG00000030551	Nr2f2	0,004272	2,11
ENSMUSG00000041734	Kirrel	0,000181	2,10
ENSMUSG00000031963	Bmper	0,002233	2,10
ENSMUSG00000034675	Dbn1	0,000076	2,10
ENSMUSG00000020897	Aurkb	0,000137	2,10
ENSMUSG00000051934	Spats2	0,000762	2,10
ENSMUSG00000026586	Prrx1	0,000262	2,09
ENSMUSG00000069920	B3gnt9	0,000018	2,09
ENSMUSG00000032194	Kank2	0,000010	2,09
ENSMUSG00000035914	Cd276	0,000005	2,08
ENSMUSG00000013076	Amotl1	0,000292	2,08
ENSMUSG00000005410	Mcm5	0,000025	2,08
ENSMUSG00000007783	Cpt1c	0,002208	2,08
ENSMUSG00000028541	B4galt2	0,003629	2,07
ENSMUSG00000054555	Adam12	0,000137	2,07
ENSMUSG00000020303	Stc2	0,001211	2,07
ENSMUSG00000050212	Eva1b	0,000050	2,06
ENSMUSG00000019966	Kitl	0,000234	2,06
ENSMUSG00000039115	Itga9	0,000027	2,06
ENSMUSG00000046798	Cldn12	0,000050	2,06
ENSMUSG00000027254	Map1a	0,000445	2,05
ENSMUSG00000029090	Adgra3	0,001267	2,05
ENSMUSG00000026355	Mcm6	0,000002	2,05
ENSMUSG00000024074	Crim1	0,000258	2,05
ENSMUSG00000017386	Traf4	0,000376	2,05
ENSMUSG00000017146	Brca1	0,000981	2,05
ENSMUSG00000032352	Lrrc1	0,004976	2,05
ENSMUSG00000017861	Mybl2	0,004374	2,04
ENSMUSG00000054702	Ap1s3	0,003340	2,04
ENSMUSG00000022754	Tmem45a	0,001480	2,04
ENSMUSG00000021782	Dlg5	0,001667	2,04
ENSMUSG00000020092	Pald1	0,000572	2,04

ENSMUSG00000030852	Tacc2	0,000072	2,04
ENSMUSG00000041949	Tango6	0,003954	2,04
ENSMUSG00000032332	Col12a1	0,001678	2,03
ENSMUSG00000078350	Smim1	0,000001	2,03
ENSMUSG00000031486	Adgra2	0,000043	2,02
ENSMUSG00000041120	Nbl1	0,000364	2,02
ENSMUSG00000027797	Dclk1	0,000229	2,02
ENSMUSG00000022995	Enah	0,001199	2,02
ENSMUSG00000052534	Pbx1	0,000004	2,02
ENSMUSG00000043587	Pxylp1	0,001657	2,02
ENSMUSG00000036545	Adamts2	0,000103	2,01
ENSMUSG00000008090	Fgfrl1	0,002164	2,01
ENSMUSG00000034371	Tkfc	0,000054	2,01
ENSMUSG00000026490	Cdc42bpa	0,000301	2,01
ENSMUSG00000032363	Adamts7	0,000243	2,00
ENSMUSG00000075592	Nynrin	0,000114	2,00
ENSMUSG00000052397	Ezr	0,000001	2,00
ENSMUSG00000026558	Uck2	0,000084	1,99
ENSMUSG00000066456	Hmgn3	0,002758	1,99
ENSMUSG00000002504	Slc9a3r2	0,000269	1,99
ENSMUSG00000001036	Epn2	0,000302	1,99
ENSMUSG00000028597	Gpx7	0,001192	1,98
ENSMUSG00000049723	Mmp12	0,000000	1,98
ENSMUSG00000036019	Tmtc2	0,003962	1,98
ENSMUSG00000046432	Bex3	0,000064	1,98
ENSMUSG00000014303	Glis2	0,001693	1,98
ENSMUSG00000002944	Cd36	0,000000	1,98
ENSMUSG00000037999	Arap2	0,000814	1,98
ENSMUSG00000033031	C330027C09Rik	0,001606	1,97
ENSMUSG00000032366	Tpm1	0,000010	1,97
ENSMUSG00000079553	Kifc1	0,001416	1,97
ENSMUSG00000021867	Tmem254b	0,000174	1,97
ENSMUSG00000072680	Tmem254c	0,000232	1,97
ENSMUSG00000029781	Fkbp9	0,000002	1,96
ENSMUSG00000027204	Fbn1	0,000566	1,96
ENSMUSG00000022385	Gtse1	0,004272	1,96
ENSMUSG00000024501	Dpysl3	0,000558	1,96
ENSMUSG00000023348	Trip6	0,000352	1,96
ENSMUSG00000024791	Cdca5	0,002417	1,96
ENSMUSG00000049791	Fzd4	0,001856	1,95
ENSMUSG00000030410	Dmwd	0,001434	1,95
ENSMUSG00000033542	Arhgef5	0,001392	1,95
ENSMUSG00000033955	Tnks1bp1	0,000020	1,95
ENSMUSG00000020099	Unc5b	0,000106	1,95
ENSMUSG00000020689	Itgb3	0,000454	1,94
ENSMUSG00000025151	Maged1	0,000013	1,93
ENSMUSG00000072676	Tmem254a	0,000392	1,93

ENSMUSG00000068744	Psrc1	0,001416	1,93
ENSMUSG00000053644	Aldh7a1	0,000093	1,93
ENSMUSG00000031697	Orc6	0,000596	1,93
ENSMUSG00000021215	Net1	0,002016	1,92
ENSMUSG00000005124	Wisp1	0,003508	1,92
ENSMUSG00000068220	Lgals1	0,000000	1,92
ENSMUSG00000031004	Mki67	0,000139	1,92
ENSMUSG00000029730	Mcm7	0,000073	1,92
ENSMUSG00000029632	Ndufa4	0,000000	1,91
ENSMUSG00000044813	Shb	0,000279	1,91
ENSMUSG00000021759	Plpp1	0,000043	1,91
ENSMUSG00000042821	Snai1	0,004610	1,91
ENSMUSG00000030516	Tjp1	0,004457	1,91
ENSMUSG00000032470	Mras	0,000046	1,91
ENSMUSG00000076431	Sox4	0,002916	1,91
ENSMUSG00000001911	Nfix	0,000137	1,91
ENSMUSG00000026575	Nme7	0,000554	1,90
ENSMUSG00000030782	Tgfb1i1	0,001690	1,90
ENSMUSG00000001506	Col1a1	0,000091	1,90
ENSMUSG00000024924	Vldlr	0,003750	1,90
ENSMUSG00000028980	H6pd	0,000002	1,89
ENSMUSG00000026817	Ak1	0,000141	1,89
ENSMUSG00000019975	Ikbip	0,000005	1,88
ENSMUSG00000027597	Ahcy	0,000001	1,87
ENSMUSG00000022791	Tnk2	0,000478	1,87
ENSMUSG00000029185	Fam114a1	0,000024	1,87
ENSMUSG00000079685	Ulbp1	0,002093	1,87
ENSMUSG00000025574	Tk1	0,000659	1,86
ENSMUSG00000039960	Rhou	0,000284	1,85
ENSMUSG00000006442	Srm	0,000049	1,85
ENSMUSG00000038244	Mical2	0,000591	1,85
ENSMUSG00000045328	Cenpe	0,004842	1,85
ENSMUSG00000032418	Me1	0,001029	1,85
ENSMUSG00000000184	Ccnd2	0,003549	1,85
ENSMUSG00000022562	Oplah	0,002062	1,84
ENSMUSG00000013033	Adgrl1	0,002943	1,84
ENSMUSG00000021823	Vcl	0,000020	1,84
ENSMUSG00000024909	Efemp2	0,000056	1,83
ENSMUSG00000005397	Nid1	0,001569	1,82
ENSMUSG00000028082	Sh3d19	0,000889	1,82
ENSMUSG00000025007	Aldh18a1	0,000109	1,82
ENSMUSG00000020363	Gfpt2	0,004600	1,81
ENSMUSG00000039043	Arpin	0,003734	1,81
ENSMUSG00000047414	Flrt2	0,000103	1,81
ENSMUSG00000040010	Slc7a5	0,002748	1,80
ENSMUSG00000032491	Nradd	0,001111	1,80
ENSMUSG00000005973	Rcn1	0,000079	1,80

ENSMUSG00000028312	Smc2	0,001208	1,78
ENSMUSG00000021477	Ctsl	0,000894	1,78
ENSMUSG00000002870	Mcm2	0,000217	1,78
ENSMUSG00000041313	Slc7a1	0,000713	1,78
ENSMUSG00000079111	Kdelr2	0,000001	1,77
ENSMUSG00000038217	Tlcd2	0,002284	1,77
ENSMUSG00000029910	Mad2l1	0,000258	1,77
ENSMUSG00000036533	Cdc42ep3	0,000679	1,77
ENSMUSG00000015568	Lpl	0,000000	1,76
ENSMUSG00000026837	Col5a1	0,000787	1,76
ENSMUSG00000046841	Ckap4	0,000000	1,75
ENSMUSG00000024098	Twsg1	0,000063	1,75
ENSMUSG00000040618	Pck2	0,000074	1,74
ENSMUSG00000029304	Spp1	0,000535	1,74
ENSMUSG00000031788	Kifc3	0,000693	1,74
ENSMUSG00000037313	Tacc3	0,001212	1,74
ENSMUSG00000058756	Thra	0,000674	1,73
ENSMUSG00000032902	Slc16a1	0,000217	1,73
ENSMUSG00000039221	Rpl22l1	0,000035	1,73
ENSMUSG00000028565	Nfia	0,003131	1,73
ENSMUSG00000020986	Sec23a	0,001273	1,73
ENSMUSG00000025268	Maged2	0,000466	1,73
ENSMUSG00000058056	Palld	0,000515	1,73
ENSMUSG00000037071	Scd1	0,001458	1,73
ENSMUSG00000029860	Zyx	0,000003	1,72
ENSMUSG00000038991	Txndc5	0,000000	1,72
ENSMUSG00000026411	Tmem9	0,002235	1,72
ENSMUSG00000037656	Slc20a2	0,001001	1,71
ENSMUSG00000022098	Bmp1	0,002306	1,71
ENSMUSG00000021238	Aldh6a1	0,001318	1,71
ENSMUSG00000038252	Ncapd2	0,001463	1,71
ENSMUSG00000040681	Hmgn1	0,000003	1,71
ENSMUSG00000057497	Fam136a	0,000599	1,70
ENSMUSG00000049288	Lix1l	0,000295	1,70
ENSMUSG00000034349	Smc4	0,000418	1,70
ENSMUSG00000047409	Ctdspl	0,000812	1,70
ENSMUSG00000025510	Cd151	0,000013	1,70
ENSMUSG00000022346	Myc	0,002501	1,69
ENSMUSG00000032047	Acat1	0,000719	1,69
ENSMUSG00000039234	Sec24d	0,000594	1,69
ENSMUSG00000029177	Cenpa	0,001458	1,69
ENSMUSG00000049932	H2afx	0,000325	1,68
ENSMUSG00000020108	Ddit4	0,002906	1,68
ENSMUSG00000003779	Kif20a	0,001579	1,68
ENSMUSG00000062825	Actg1	0,000000	1,68
ENSMUSG00000030319	Cand2	0,000338	1,68
ENSMUSG00000032324	Tspan3	0,000057	1,68

ENSMUSG00000021756	Il6st	0,000176	1,67
ENSMUSG00000029767	Calu	0,000000	1,67
ENSMUSG00000040084	Bub1b	0,003765	1,67
ENSMUSG00000061838	Suclg2	0,000708	1,67
ENSMUSG00000035376	Hacd2	0,001068	1,66
ENSMUSG00000027602	Map1lc3a	0,003460	1,66
ENSMUSG00000021556	Golm1	0,000362	1,65
ENSMUSG00000029373	Pf4	0,004009	1,65
ENSMUSG00000024048	Myl12a	0,000000	1,65
ENSMUSG00000006373	Pgrmc1	0,000059	1,64
ENSMUSG00000026421	Csrp1	0,000141	1,64
ENSMUSG00000026335	Pam	0,000160	1,64
ENSMUSG00000032518	Rpsa	0,000000	1,64
ENSMUSG00000035342	Lzts2	0,003107	1,64
ENSMUSG00000029762	Akr1b8	0,002237	1,64
ENSMUSG00000026193	Fn1	0,003576	1,64
ENSMUSG00000023022	Lima1	0,000040	1,63
ENSMUSG00000019122	Ccl9	0,003967	1,63
ENSMUSG00000027488	Snta1	0,003318	1,63
ENSMUSG00000021811	Dnajc9	0,003472	1,62
ENSMUSG00000011179	Odc1	0,001412	1,62
ENSMUSG00000047250	Ptgs1	0,002727	1,61
ENSMUSG00000026509	Capn2	0,000242	1,61
ENSMUSG00000022863	Btg3	0,002845	1,61
ENSMUSG00000008668	Rps18	0,000000	1,60
ENSMUSG00000022010	Tsc22d1	0,000745	1,60
ENSMUSG00000038900	Rpl12	0,000000	1,60
ENSMUSG00000044783	Hjurp	0,003375	1,60
ENSMUSG00000033863	Klf9	0,003037	1,60
ENSMUSG00000039952	Dag1	0,000479	1,59
ENSMUSG00000034118	Tpst1	0,002351	1,59
ENSMUSG00000030879	Mrpl17	0,001110	1,59
ENSMUSG00000091337	Eid1	0,001170	1,59
ENSMUSG00000031353	Rbbp7	0,000523	1,59
ENSMUSG00000018362	Kpna2	0,000393	1,58
ENSMUSG00000038416	Cdc16	0,001371	1,58
ENSMUSG00000041355	Ssr2	0,000477	1,58
ENSMUSG00000031799	Tpm4	0,000000	1,58
ENSMUSG00000029250	Polr2b	0,000734	1,58
ENSMUSG00000021703	Serinc5	0,004228	1,58
ENSMUSG00000027907	S100a11	0,000491	1,57
ENSMUSG00000024165	Hn1l	0,004372	1,57
ENSMUSG00000021824	Ap3m1	0,000206	1,57
ENSMUSG00000057841	Rpl32	0,000478	1,56
ENSMUSG00000027828	Ssr3	0,000116	1,56
ENSMUSG00000016946	Kctd5	0,001496	1,56
ENSMUSG00000030541	Idh2	0,000644	1,55

ENSMUSG00000012405	Rpl15	0,000000	1,55
ENSMUSG00000024659	Anxa1	0,001424	1,54
ENSMUSG00000031146	Plp2	0,004275	1,53
ENSMUSG00000033769	Exoc6b	0,004691	1,53
ENSMUSG00000015759	Cnih1	0,001466	1,53
ENSMUSG00000001473	Tubb6	0,000747	1,53
ENSMUSG00000040652	Oaz2	0,002000	1,53
ENSMUSG00000037805	Rpl10a	0,000000	1,52
ENSMUSG00000025967	Eef1b2	0,000466	1,52
ENSMUSG00000029777	Gars	0,002344	1,51
ENSMUSG00000044533	Rps2	0,000000	1,51
ENSMUSG00000060860	Ube2s	0,001119	1,51
ENSMUSG00000023067	Cdkn1a	0,004272	1,51
ENSMUSG00000008393	Carhsp1	0,002119	1,50
ENSMUSG00000005732	Ranbp1	0,005061	1,50
ENSMUSG00000019790	Stxbp5	0,002906	-1,50
ENSMUSG00000020863	Luc7l3	0,004311	-1,50
ENSMUSG00000026339	Ccdc93	0,002525	-1,50
ENSMUSG00000034116	Vav1	0,002738	-1,50
ENSMUSG00000024818	Slc25a45	0,004028	-1,50
ENSMUSG00000024858	Grk2	0,000020	-1,51
ENSMUSG00000032295	Man2c1	0,001992	-1,51
ENSMUSG00000039512	Uhrf1bp1	0,003299	-1,51
ENSMUSG00000027878	Notch2	0,002953	-1,51
ENSMUSG00000031537	Ikbkb	0,000372	-1,51
ENSMUSG00000027639	Samhd1	0,003208	-1,51
ENSMUSG00000031808	Slc27a1	0,002147	-1,51
ENSMUSG00000056917	Sipa1	0,000973	-1,51
ENSMUSG00000024045	Akap8	0,002109	-1,51
ENSMUSG00000034312	Iqsec1	0,000000	-1,51
ENSMUSG00000026425	Srgap2	0,003594	-1,51
ENSMUSG00000030720	Cln3	0,001832	-1,51
ENSMUSG00000020053	Igf1	0,003982	-1,51
ENSMUSG00000051344	Plekhm3	0,003616	-1,52
ENSMUSG00000078945	Naip2	0,004705	-1,52
ENSMUSG00000034160	Ogt	0,001688	-1,52
ENSMUSG00000048865	Arhgap30	0,000051	-1,52
ENSMUSG00000074785	Plxnc1	0,003655	-1,52
ENSMUSG00000047153	Khnyn	0,004055	-1,52
ENSMUSG00000034022	Cpsf1	0,003649	-1,52
ENSMUSG00000029098	Acox3	0,000010	-1,52
ENSMUSG00000071715	Ncf4	0,000879	-1,52
ENSMUSG00000030287	Itpr2	0,005081	-1,52
ENSMUSG00000051495	Irf2bp2	0,000280	-1,52
ENSMUSG00000020994	Pnn	0,004228	-1,52
ENSMUSG00000027399	Il1a	0,002501	-1,52
ENSMUSG00000034163	Zfc3h1	0,004490	-1,52

ENSMUSG00000047407	Tgif1	0,000673	-1,52
ENSMUSG00000030122	Ptms	0,002370	-1,53
ENSMUSG00000047098	Rnf31	0,004507	-1,53
ENSMUSG00000026288	Inpp5d	0,001673	-1,53
ENSMUSG00000004626	Stxbp2	0,001854	-1,53
ENSMUSG00000022952	Runx1	0,004511	-1,53
ENSMUSG00000046556	Zfp319	0,003281	-1,53
ENSMUSG00000025153	Fasn	0,003914	-1,53
ENSMUSG00000020385	Clk4	0,003682	-1,53
ENSMUSG00000041836	Ptpre	0,000427	-1,53
ENSMUSG00000039936	Pik3cd	0,002089	-1,53
ENSMUSG00000022436	Sh3bp1	0,001673	-1,54
ENSMUSG00000051506	Wdfy4	0,004554	-1,54
ENSMUSG00000062082	Cd200r4	0,001739	-1,54
ENSMUSG00000022831	Hcls1	0,004391	-1,54
ENSMUSG00000037860	Aim2	0,002727	-1,54
ENSMUSG00000014353	Tmem87b	0,001466	-1,54
ENSMUSG00000036054	Sugp2	0,004275	-1,54
ENSMUSG00000032633	Flcn	0,001247	-1,54
ENSMUSG00000000275	Trim25	0,000000	-1,54
ENSMUSG00000024052	Lpin2	0,001136	-1,54
ENSMUSG00000026113	Inpp4a	0,001038	-1,54
ENSMUSG00000075595	Zfp652	0,001646	-1,55
ENSMUSG00000045917	Tmem268	0,001414	-1,55
ENSMUSG00000031133	Arhgef6	0,001185	-1,55
ENSMUSG00000048249	Crebrf	0,003770	-1,55
ENSMUSG00000026471	Mr1	0,001730	-1,55
ENSMUSG00000008200	Fnbp4	0,001273	-1,55
ENSMUSG00000020275	Rel	0,002717	-1,55
ENSMUSG00000000628	Hk2	0,000873	-1,55
ENSMUSG00000042404	Dennd4b	0,000523	-1,55
ENSMUSG00000021457	Syk	0,000000	-1,55
ENSMUSG00000023191	P3h3	0,002760	-1,55
ENSMUSG00000029428	Stx2	0,001049	-1,56
ENSMUSG00000026395	Ptprc	0,001950	-1,56
ENSMUSG00000020740	Gga3	0,000552	-1,56
ENSMUSG00000032089	Il10ra	0,003597	-1,56
ENSMUSG00000028793	Rnf19b	0,003655	-1,56
ENSMUSG00000041328	Pcf11	0,003294	-1,56
ENSMUSG00000046805	Mpeg1	0,000013	-1,56
ENSMUSG00000000441	Raf1	0,000077	-1,56
ENSMUSG00000028649	Macf1	0,001429	-1,56
ENSMUSG00000052512	Nav2	0,001847	-1,56
ENSMUSG00000089917	Uckl1	0,001466	-1,56
ENSMUSG00000038271	Iffo1	0,002208	-1,56
ENSMUSG00000044456	Rin3	0,004276	-1,57
ENSMUSG00000020806	Rhbdf2	0,000481	-1,57

ENSMUSG00000034647	Ankrd12	0,002471	-1,57
ENSMUSG00000022372	Sla	0,004492	-1,57
ENSMUSG00000020387	Jade2	0,002580	-1,57
ENSMUSG00000034792	Gna15	0,002752	-1,57
ENSMUSG00000027522	Stx16	0,000864	-1,57
ENSMUSG00000042444	Fam63b	0,001066	-1,57
ENSMUSG00000021886	Gpr65	0,004973	-1,58
ENSMUSG00000021948	Prkcd	0,000000	-1,58
ENSMUSG00000038179	Slamf7	0,000746	-1,58
ENSMUSG00000033253	Szt2	0,002187	-1,58
ENSMUSG00000032119	Hinfp	0,003550	-1,58
ENSMUSG00000039637	Coro7	0,000480	-1,58
ENSMUSG00000047909	Ankrd16	0,002392	-1,58
ENSMUSG00000029647	Pan3	0,000851	-1,58
ENSMUSG00000030761	Myo7a	0,000728	-1,58
ENSMUSG00000030084	Plxna1	0,000000	-1,58
ENSMUSG00000031709	Tbc1d9	0,000442	-1,58
ENSMUSG00000031166	Wdr13	0,000695	-1,58
ENSMUSG00000025137	Pcyt2	0,001337	-1,58
ENSMUSG00000025949	Pikfyve	0,000565	-1,59
ENSMUSG00000036986	Pml	0,002269	-1,59
ENSMUSG00000022974	Paxbp1	0,002089	-1,59
ENSMUSG00000036552	Ermard	0,000796	-1,59
ENSMUSG00000041935	AW549877	0,001709	-1,59
ENSMUSG00000018707	Dync1h1	0,000740	-1,59
ENSMUSG00000025856	Pdgfa	0,003762	-1,59
ENSMUSG00000060216	Arrb2	0,000536	-1,59
ENSMUSG00000033450	Tagap	0,002484	-1,59
ENSMUSG00000023830	Igf2r	0,000115	-1,59
ENSMUSG00000043940	Wdfy3	0,004962	-1,59
ENSMUSG00000055024	Ep300	0,004234	-1,59
ENSMUSG00000040943	Tet2	0,002382	-1,60
ENSMUSG00000042599	Kdm7a	0,002343	-1,60
ENSMUSG00000015305	Sash1	0,001901	-1,60
ENSMUSG00000005686	Ampd3	0,000711	-1,60
ENSMUSG00000028842	Ago3	0,001809	-1,60
ENSMUSG00000052331	Ankrd44	0,003131	-1,60
ENSMUSG00000033004	Mycbp2	0,003341	-1,60
ENSMUSG00000002897	Il17ra	0,000392	-1,60
ENSMUSG00000002028	Kmt2a	0,001305	-1,61
ENSMUSG00000061411	Nol4l	0,003888	-1,61
ENSMUSG00000040268	Plekha1	0,000001	-1,61
ENSMUSG00000025969	Nrp2	0,000720	-1,61
ENSMUSG00000032525	Nktr	0,000760	-1,61
ENSMUSG00000030256	Bhlhe41	0,000000	-1,61
ENSMUSG00000029389	Ddx55	0,004747	-1,61
ENSMUSG00000034255	Arhgap27	0,003656	-1,61

ENSMUSG00000037652	Phc3	0,002706	-1,61
ENSMUSG00000029714	Gigyf1	0,000512	-1,61
ENSMUSG00000038175	Mylip	0,003384	-1,61
ENSMUSG00000073490	Ifi207	0,000811	-1,61
ENSMUSG00000093803	Ppp2r3d	0,001211	-1,61
ENSMUSG00000032637	Atxn2l	0,000181	-1,61
ENSMUSG00000041164	Zmiz2	0,000261	-1,62
ENSMUSG00000038056	Kmt2c	0,004487	-1,62
ENSMUSG00000042423	Fbrs	0,000197	-1,62
ENSMUSG00000026500	Cox20	0,003614	-1,62
ENSMUSG00000024137	E4f1	0,002798	-1,62
ENSMUSG00000036246	Gmip	0,000238	-1,62
ENSMUSG00000020828	Pld2	0,000269	-1,62
ENSMUSG00000002910	Arrdc2	0,004355	-1,63
ENSMUSG00000028337	Coro2a	0,001247	-1,63
ENSMUSG00000006019	Dhx34	0,001104	-1,63
ENSMUSG00000078484	Klhl17	0,000921	-1,63
ENSMUSG00000001248	Gramd1a	0,000434	-1,63
ENSMUSG00000021271	Zfp839	0,002679	-1,63
ENSMUSG00000024953	Prdx5	0,002866	-1,63
ENSMUSG00000030589	Rasgrp4	0,004502	-1,63
ENSMUSG00000042675	Ypel3	0,000907	-1,64
ENSMUSG00000041695	Kcnj2	0,000624	-1,64
ENSMUSG00000027677	Ttc14	0,001690	-1,64
ENSMUSG00000069833	Ahnak	0,000178	-1,64
ENSMUSG00000003123	Lipe	0,004307	-1,64
ENSMUSG00000041515	Irf8	0,000270	-1,64
ENSMUSG00000020573	Pik3cg	0,001809	-1,64
ENSMUSG00000021583	Erap1	0,001312	-1,64
ENSMUSG00000054203	Ifi205	0,002370	-1,65
ENSMUSG00000040687	Madd	0,000243	-1,65
ENSMUSG00000025375	Aatk	0,000141	-1,65
ENSMUSG00000032024	Clmp	0,000659	-1,65
ENSMUSG00000022105	Rb1	0,000194	-1,65
ENSMUSG00000010358	Ifi35	0,000893	-1,65
ENSMUSG00000034330	Plcg2	0,000352	-1,65
ENSMUSG00000003283	Hck	0,002612	-1,65
ENSMUSG00000027615	Hps3	0,001021	-1,66
ENSMUSG00000039908	Slc26a11	0,001682	-1,66
ENSMUSG00000006517	Mvd	0,000212	-1,66
ENSMUSG00000026536	Ifi211	0,001199	-1,66
ENSMUSG00000037816	Fbxw17	0,002012	-1,66
ENSMUSG00000027829	Ccnl1	0,000264	-1,66
ENSMUSG00000066036	Ubr4	0,000231	-1,66
ENSMUSG00000044350	Lacc1	0,000255	-1,66
ENSMUSG00000049130	C5ar1	0,002048	-1,66
ENSMUSG00000035284	Vps13c	0,003909	-1,66

ENSMUSG00000024193	Phf1	0,001744	-1,66
ENSMUSG00000020893	Per1	0,000388	-1,66
ENSMUSG00000025409	Mbd6	0,000109	-1,67
ENSMUSG00000021846	Peli2	0,003973	-1,67
ENSMUSG00000041132	N4bp2l1	0,001015	-1,67
ENSMUSG00000026349	Ccnt2	0,000268	-1,67
ENSMUSG00000072812	Ahnak2	0,001417	-1,67
ENSMUSG00000039531	Zufsp	0,002044	-1,67
ENSMUSG00000030577	Cd22	0,000019	-1,67
ENSMUSG00000002325	Irf9	0,000231	-1,67
ENSMUSG00000044037	Als2cl	0,004307	-1,67
ENSMUSG00000097405	D630044L22Rik	0,000669	-1,68
ENSMUSG00000038241	Cep250	0,000171	-1,68
ENSMUSG00000039205	Ciz1	0,000121	-1,68
ENSMUSG00000087370	Tmem170b	0,000517	-1,68
ENSMUSG00000024339	Tap2	0,000222	-1,68
ENSMUSG00000038811	Gngt2	0,000463	-1,68
ENSMUSG00000072620	Slfn2	0,000364	-1,68
ENSMUSG00000005682	Pan2	0,003195	-1,68
ENSMUSG00000027009	Itga4	0,003211	-1,68
ENSMUSG00000006411	Nectin4	0,001315	-1,69
ENSMUSG00000026656	Fcgr2b	0,000264	-1,69
ENSMUSG00000030590	Fam98c	0,001993	-1,69
ENSMUSG00000013846	St3gal1	0,000807	-1,69
ENSMUSG00000044712	Slc38a6	0,000119	-1,69
ENSMUSG00000010751	Tnfrsf22	0,002608	-1,69
ENSMUSG00000006281	Tep1	0,000115	-1,69
ENSMUSG00000038664	Herc1	0,000197	-1,69
ENSMUSG00000038895	Zfp653	0,003535	-1,69
ENSMUSG00000038352	Arl5c	0,000865	-1,69
ENSMUSG00000041354	Rgl2	0,000472	-1,69
ENSMUSG00000036622	Atp13a2	0,000000	-1,69
ENSMUSG00000004565	Pnpla6	0,000141	-1,69
ENSMUSG00000022439	Parvg	0,000295	-1,70
ENSMUSG00000015243	Abca1	0,000794	-1,70
ENSMUSG00000040209	Zfp704	0,000104	-1,70
ENSMUSG00000025241	Fyco1	0,000376	-1,70
ENSMUSG00000043432	Leng9	0,002024	-1,70
ENSMUSG00000004730	Adgre1	0,000745	-1,70
ENSMUSG00000036067	Slc2a6	0,000064	-1,70
ENSMUSG00000016496	Cd274	0,000167	-1,70
ENSMUSG00000039218	Srrm2	0,000229	-1,71
ENSMUSG00000030337	Vamp1	0,001121	-1,71
ENSMUSG00000036833	Pnpla7	0,000050	-1,71
ENSMUSG00000020894	Vamp2	0,000044	-1,71
ENSMUSG00000039899	Fgl2	0,001806	-1,71
ENSMUSG00000040415	Dtx3	0,000497	-1,72

ENSMUSG00000040061	Plcb2	0,000227	-1,72
ENSMUSG00000001930	Vwf	0,000203	-1,72
ENSMUSG00000021495	Fam193b	0,000270	-1,72
ENSMUSG00000052477	C130026I21Rik	0,001087	-1,72
ENSMUSG00000034771	Tle2	0,002113	-1,72
ENSMUSG00000066152	Slc31a2	0,000028	-1,72
ENSMUSG00000023913	Pla2g7	0,000368	-1,72
ENSMUSG00000027660	Skil	0,000224	-1,73
ENSMUSG00000022489	Pde1b	0,002280	-1,73
ENSMUSG00000056130	Ticam2	0,001344	-1,73
ENSMUSG00000034616	Ssh3	0,000018	-1,73
ENSMUSG00000062515	Fabp4	0,000284	-1,73
ENSMUSG00000035967	Ints6l	0,000224	-1,73
ENSMUSG00000031442	Mcf2l	0,003883	-1,73
ENSMUSG00000043008	Klhl6	0,000726	-1,73
ENSMUSG00000017631	Abr	0,000000	-1,73
ENSMUSG00000059851	Kmt5c	0,000198	-1,73
ENSMUSG00000001062	Vps9d1	0,000059	-1,73
ENSMUSG00000066440	Zfyve26	0,000000	-1,74
ENSMUSG00000036948	BC037034	0,000050	-1,74
ENSMUSG00000021676	Iqgap2	0,000764	-1,74
ENSMUSG00000014361	Mertk	0,000096	-1,74
ENSMUSG00000046157	Tmem229b	0,000788	-1,74
ENSMUSG00000035722	Abca7	0,000030	-1,74
ENSMUSG00000047798	Cd300lf	0,000016	-1,74
ENSMUSG00000041642	Kif21b	0,000024	-1,74
ENSMUSG00000026031	Cflar	0,000019	-1,74
ENSMUSG00000078485	Plekhn1	0,000514	-1,75
ENSMUSG00000020017	Hal	0,000000	-1,75
ENSMUSG00000035697	Hmha1	0,000002	-1,75
ENSMUSG00000036478	Btg1	0,000102	-1,75
ENSMUSG00000070327	Rnf213	0,001054	-1,75
ENSMUSG00000073678	Pgap1	0,000198	-1,75
ENSMUSG00000037326	Capn15	0,000005	-1,75
ENSMUSG00000079487	Med12	0,000746	-1,76
ENSMUSG00000027508	Pag1	0,000425	-1,76
ENSMUSG00000024851	Pitpnm1	0,000011	-1,76
ENSMUSG00000024679	Ms4a6d	0,000541	-1,76
ENSMUSG00000039852	Rere	0,000000	-1,76
ENSMUSG00000042625	Safb2	0,000032	-1,77
ENSMUSG00000057894	Zfp329	0,001658	-1,77
ENSMUSG00000021640	Naip1	0,003448	-1,77
ENSMUSG00000004266	Ptpn6	0,000137	-1,77
ENSMUSG00000052142	Rasal3	0,000024	-1,77
ENSMUSG00000074794	Arrdc3	0,000889	-1,77
ENSMUSG00000046207	Pik3r6	0,001093	-1,77
ENSMUSG00000057110	Cntrl	0,000244	-1,77

ENSMUSG00000042408	Zmym6	0,001595	-1,77
ENSMUSG00000021423	Ly86	0,001378	-1,77
ENSMUSG00000078153	Psme2b	0,004096	-1,78
ENSMUSG00000038538	Ubn2	0,000032	-1,78
ENSMUSG00000001348	Acp5	0,000195	-1,78
ENSMUSG00000020709	Adap2	0,000703	-1,78
ENSMUSG00000040721	Zfhx2	0,000432	-1,78
ENSMUSG00000040712	Camta2	0,000000	-1,78
ENSMUSG00000042613	Pbxip1	0,000005	-1,78
ENSMUSG00000035164	Zc3h12c	0,002899	-1,79
ENSMUSG00000063268	Parp10	0,000026	-1,79
ENSMUSG00000074519	Zfp971	0,004629	-1,79
ENSMUSG00000039158	Akna	0,000051	-1,79
ENSMUSG00000055782	Abcd2	0,000327	-1,79
ENSMUSG00000039199	Zdhhc1	0,000245	-1,80
ENSMUSG00000054517	Trim65	0,000423	-1,80
ENSMUSG00000028248	Pnisr	0,000052	-1,80
ENSMUSG00000040327	Cul9	0,000036	-1,80
ENSMUSG00000031805	Jak3	0,000114	-1,80
ENSMUSG00000026928	Card9	0,000022	-1,80
ENSMUSG00000039621	Prex1	0,000006	-1,80
ENSMUSG00000073411	H2-D1	0,000047	-1,81
ENSMUSG00000021298	Gpr132	0,000870	-1,81
ENSMUSG00000019970	Sgk1	0,000000	-1,81
ENSMUSG00000057143	Trim12c	0,000036	-1,81
ENSMUSG00000037266	Rsrp1	0,000004	-1,81
ENSMUSG00000038608	Dock10	0,000002	-1,81
ENSMUSG00000049191	Rgag4	0,000007	-1,82
ENSMUSG00000004267	Eno2	0,000335	-1,82
ENSMUSG00000055866	Per2	0,000650	-1,82
ENSMUSG00000038213	Tapbpl	0,000784	-1,82
ENSMUSG00000036528	Ppfibp2	0,000009	-1,82
ENSMUSG00000020261	Slc36a1	0,000000	-1,83
ENSMUSG00000038151	Prdm1	0,004787	-1,83
ENSMUSG00000039109	F13a1	0,002752	-1,83
ENSMUSG00000031740	Mmp2	0,002570	-1,83
ENSMUSG00000036853	Mcoln3	0,000079	-1,83
ENSMUSG00000022822	Abcc5	0,000000	-1,83
ENSMUSG00000027950	Chrnb2	0,000011	-1,84
ENSMUSG00000048154	Kmt2d	0,000011	-1,84
ENSMUSG00000074733	Zfp950	0,000035	-1,84
ENSMUSG00000056724	Nbeal2	0,000006	-1,84
ENSMUSG00000040522	Tlr8	0,000540	-1,85
ENSMUSG00000032013	Trim29	0,000990	-1,87
ENSMUSG00000079293	Clec7a	0,000020	-1,87
ENSMUSG00000067212	H2-T23	0,000000	-1,87
ENSMUSG00000005986	Ankrd13d	0,001139	-1,88

ENSMUSG00000038437	Mllt6	0,000000	-1,88
ENSMUSG00000015942	Gtf2ird2	0,000010	-1,88
ENSMUSG00000060441	Trim5	0,003142	-1,88
ENSMUSG00000035545	Leng8	0,000005	-1,88
ENSMUSG00000030123	Plxnd1	0,000004	-1,88
ENSMUSG00000015950	Ncf1	0,000005	-1,88
ENSMUSG00000031897	Psmb10	0,000087	-1,89
ENSMUSG00000016206	H2-M3	0,000073	-1,89
ENSMUSG00000028957	Per3	0,000504	-1,89
ENSMUSG00000037997	Parp11	0,000028	-1,89
ENSMUSG00000047810	Ccdc88b	0,000001	-1,89
ENSMUSG00000056185	Snx32	0,000113	-1,89
ENSMUSG00000028076	Cd1d1	0,000152	-1,89
ENSMUSG00000110206	Flt3l	0,002629	-1,89
ENSMUSG00000038188	Scarf1	0,000027	-1,90
ENSMUSG00000027078	Ube2l6	0,000011	-1,90
ENSMUSG00000037731	Themis2	0,000031	-1,90
ENSMUSG00000020865	Abcc3	0,000001	-1,90
ENSMUSG00000032589	Bsn	0,000182	-1,91
ENSMUSG00000041936	Agrn	0,000028	-1,91
ENSMUSG00000042349	Ikbke	0,000000	-1,91
ENSMUSG00000016495	Plgrkt	0,000005	-1,91
ENSMUSG00000020263	Appl2	0,000001	-1,92
ENSMUSG00000023764	Sfi1	0,000091	-1,93
ENSMUSG00000041775	Mapk1ip1	0,000042	-1,93
ENSMUSG00000005800	Mmp8	0,000472	-1,93
ENSMUSG00000053158	Fes	0,000000	-1,93
ENSMUSG00000025586	Cpeb1	0,000007	-1,94
ENSMUSG00000098188	Sowahc	0,000000	-1,94
ENSMUSG00000023473	Celsr3	0,000050	-1,94
ENSMUSG00000022014	Epsti1	0,003219	-1,94
ENSMUSG00000049037	Clec4a1	0,002021	-1,94
ENSMUSG00000006818	Sod2	0,000065	-1,94
ENSMUSG00000000982	Ccl3	0,000000	-1,94
ENSMUSG00000033781	Asb13	0,000052	-1,95
ENSMUSG00000078486	Perm1	0,000055	-1,95
ENSMUSG00000067714	Lpar5	0,000901	-1,95
ENSMUSG00000027676	Ccdc39	0,001770	-1,95
ENSMUSG00000024174	Pot1b	0,000364	-1,95
ENSMUSG00000020023	Tmcc3	0,000031	-1,95
ENSMUSG00000027951	Adar	0,000000	-1,96
ENSMUSG00000044583	Tlr7	0,000003	-1,96
ENSMUSG00000039395	Mreg	0,000010	-1,96
ENSMUSG00000078853	Igtp	0,000061	-1,96
ENSMUSG00000004952	Rasa4	0,000000	-1,97
ENSMUSG00000056592	Zfp658	0,000659	-1,97
ENSMUSG00000042129	Rassf4	0,000039	-1,97

ENSMUSG00000024401	Tnf	0,000005	-1,97
ENSMUSG00000038507	Parp12	0,000018	-1,98
ENSMUSG00000072720	Myo18b	0,000233	-1,98
ENSMUSG00000026896	Ifih1	0,000047	-1,98
ENSMUSG00000078942	Naip6	0,000001	-1,98
ENSMUSG00000001053	N4bp3	0,000271	-1,98
ENSMUSG00000070390	Nlrp1b	0,000410	-1,99
ENSMUSG00000039853	Trim14	0,000777	-1,99
ENSMUSG00000022148	Fyb	0,000068	-1,99
ENSMUSG00000024403	Atp6v1g2	0,000600	-1,99
ENSMUSG00000026814	Eng	0,000001	-2,00
ENSMUSG00000033705	Stard9	0,000006	-2,00
ENSMUSG00000043243	Fam129c	0,001321	-2,01
ENSMUSG00000036273	Lrrk2	0,000018	-2,01
ENSMUSG00000026104	Stat1	0,000014	-2,02
ENSMUSG00000054404	Slfn5	0,000000	-2,03
ENSMUSG00000045875	Adra1a	0,000017	-2,03
ENSMUSG00000032691	Nlrp3	0,000225	-2,03
ENSMUSG00000076441	Ass1	0,000018	-2,03
ENSMUSG00000090215	Trim34b	0,000761	-2,03
ENSMUSG00000024621	Csf1r	0,000000	-2,03
ENSMUSG00000071203	Naip5	0,000000	-2,04
ENSMUSG00000037697	Ddhd1	0,000000	-2,04
ENSMUSG00000044340	Phlpp1	0,000039	-2,05
ENSMUSG00000031444	F10	0,000043	-2,05
ENSMUSG00000017830	Dhx58	0,000002	-2,05
ENSMUSG00000002668	Dennd1c	0,000001	-2,05
ENSMUSG00000078896	Zfp965	0,002589	-2,05
ENSMUSG00000032265	Fam46a	0,000000	-2,05
ENSMUSG00000046245	Pilra	0,000218	-2,06
ENSMUSG00000039699	Batf2	0,001110	-2,06
ENSMUSG00000056144	Trim34a	0,000015	-2,07
ENSMUSG00000030276	Ttll3	0,000184	-2,07
ENSMUSG00000095105	Edaradd	0,001568	-2,07
ENSMUSG00000041268	Dmxl2	0,000000	-2,07
ENSMUSG00000030142	Clec4e	0,000006	-2,08
ENSMUSG00000026923	Notch1	0,000001	-2,08
ENSMUSG00000026222	Sp100	0,000000	-2,08
ENSMUSG00000090675	Olfr111	0,000296	-2,08
ENSMUSG00000090894	Olfr110	0,003745	-2,09
ENSMUSG00000056054	S100a8	0,004342	-2,09
ENSMUSG00000038963	Slco4a1	0,000000	-2,09
ENSMUSG00000015947	Fcgr1	0,000083	-2,09
ENSMUSG00000032561	Acpp	0,000085	-2,10
ENSMUSG00000052776	Oas1a	0,000000	-2,10
ENSMUSG00000060802	B2m	0,000001	-2,10
ENSMUSG00000008496	Pou2f2	0,000007	-2,10

ENSMUSG00000037649	H2-DMa	0,000150	-2,11
ENSMUSG00000035713	Usp35	0,000000	-2,11
ENSMUSG00000036526	Card11	0,004622	-2,12
ENSMUSG00000013974	Mcemp1	0,000746	-2,12
ENSMUSG00000030921	Trim30a	0,000000	-2,12
ENSMUSG00000066258	Trim12a	0,000007	-2,13
ENSMUSG00000093445	Lrch4	0,001505	-2,14
ENSMUSG00000040829	Zmynd15	0,000000	-2,15
ENSMUSG00000028979	Masp2	0,000045	-2,15
ENSMUSG00000026875	Traf1	0,000000	-2,16
ENSMUSG00000074657	Kif5a	0,000612	-2,16
ENSMUSG00000040026	Saa3	0,000007	-2,17
ENSMUSG00000002985	Apoe	0,000000	-2,17
ENSMUSG00000030745	Il21r	0,000037	-2,18
ENSMUSG00000042249	Grk3	0,000000	-2,18
ENSMUSG00000030330	Ing4	0,004060	-2,18
ENSMUSG00000055994	Nod2	0,000111	-2,19
ENSMUSG00000024308	Tapbp	0,000000	-2,19
ENSMUSG00000024672	Ms4a7	0,000001	-2,19
ENSMUSG00000042190	Cmklr1	0,000006	-2,19
ENSMUSG00000033192	Lpcat2	0,000103	-2,20
ENSMUSG00000029298	Gbp9	0,000013	-2,20
ENSMUSG00000022906	Parp9	0,000000	-2,20
ENSMUSG00000040296	Ddx58	0,000000	-2,21
ENSMUSG00000049313	Sorl1	0,000000	-2,21
ENSMUSG00000018920	Cxcl16	0,000000	-2,22
ENSMUSG00000056116	H2-T22	0,000000	-2,23
ENSMUSG00000028064	Sema4a	0,000000	-2,23
ENSMUSG00000028327	Stra6l	0,000000	-2,23
ENSMUSG00000046718	Bst2	0,000000	-2,24
ENSMUSG00000039501	Znfx1	0,000000	-2,24
ENSMUSG00000070034	Sp110	0,000000	-2,25
ENSMUSG00000031200	Mtcp1	0,001014	-2,26
ENSMUSG00000026764	Kif5c	0,000025	-2,26
ENSMUSG00000027580	Helz2	0,000000	-2,26
ENSMUSG00000028339	Col15a1	0,000119	-2,28
ENSMUSG00000018986	Slfn3	0,001466	-2,28
ENSMUSG00000091649	Phf11b	0,000064	-2,28
ENSMUSG00000029605	Oas1b	0,000143	-2,29
ENSMUSG00000028859	Csf3r	0,000000	-2,29
ENSMUSG00000030117	Gdf3	0,000836	-2,29
ENSMUSG00000048142	Nat8l	0,000229	-2,29
ENSMUSG00000042726	Trafd1	0,000000	-2,30
ENSMUSG00000091144	Phf11c	0,000169	-2,33
ENSMUSG00000049502	Dtx3l	0,000000	-2,35
ENSMUSG00000035513	Ntng2	0,000110	-2,35
ENSMUSG00000032596	Uba7	0,000000	-2,36

ENSMUSG00000026980	Ly75	0,000000	-2,36
ENSMUSG00000015340	Cybb	0,000000	-2,37
ENSMUSG00000066682	Pilrb2	0,000112	-2,37
ENSMUSG00000021990	Spata13	0,000113	-2,37
ENSMUSG00000069516	Lyz2	0,000000	-2,37
ENSMUSG00000017390	Aldoc	0,000001	-2,37
ENSMUSG00000051650	B3gnt2	0,001483	-2,37
ENSMUSG00000018899	Irf1	0,000000	-2,38
ENSMUSG00000000732	Icosl	0,000792	-2,39
ENSMUSG00000039934	Gsap	0,000000	-2,39
ENSMUSG00000019301	Hsd17b1	0,000912	-2,39
ENSMUSG00000057596	Trim30d	0,000001	-2,39
ENSMUSG00000039431	Mtmr7	0,000685	-2,39
ENSMUSG00000090272	Mndal	0,000000	-2,40
ENSMUSG00000043017	Ptgir	0,000000	-2,40
ENSMUSG00000033446	Lpar6	0,000000	-2,40
ENSMUSG00000033871	Ppargc1b	0,000649	-2,41
ENSMUSG00000024338	Psmb8	0,000000	-2,41
ENSMUSG00000053101	Gpr141	0,000052	-2,43
ENSMUSG00000037418	Best1	0,000016	-2,44
ENSMUSG00000029442	Wdr66	0,003664	-2,46
ENSMUSG00000027318	Adam33	0,000012	-2,47
ENSMUSG00000027995	Tlr2	0,000000	-2,47
ENSMUSG00000022126	Acod1	0,000000	-2,47
ENSMUSG00000066861	Oas1g	0,000000	-2,48
ENSMUSG00000040033	Stat2	0,000000	-2,49
ENSMUSG00000096727	Psmb9	0,000000	-2,49
ENSMUSG00000053835	H2-T24	0,000000	-2,49
ENSMUSG00000038037	Socs1	0,001132	-2,51
ENSMUSG00000036661	Dennd3	0,000000	-2,52
ENSMUSG00000029490	Mfsd7a	0,000000	-2,52
ENSMUSG00000000791	Il12rb1	0,004565	-2,52
ENSMUSG00000069874	Irgm2	0,000000	-2,54
ENSMUSG00000044468	Fam46c	0,000000	-2,55
ENSMUSG00000046879	Irgm1	0,000000	-2,55
ENSMUSG00000020641	Rsad2	0,000000	-2,59
ENSMUSG00000047735	Samd9l	0,000000	-2,59
ENSMUSG00000080316	Spaca6	0,000000	-2,59
ENSMUSG00000066684	Pilrb1	0,000023	-2,62
ENSMUSG00000024245	Tmem178	0,000000	-2,63
ENSMUSG00000091705	H2-Q2	0,000000	-2,64
ENSMUSG00000029798	Herc6	0,000000	-2,64
ENSMUSG00000041324	Inhba	0,000028	-2,65
ENSMUSG00000061972	Olfr99	0,003909	-2,65
ENSMUSG00000033880	Lgals3bp	0,000000	-2,65
ENSMUSG00000068245	Phf11d	0,000000	-2,66
ENSMUSG00000001588	Acap1	0,000792	-2,66

ENSMUSG00000073406	H2-Bl	0,000002	-2,68
ENSMUSG00000039997	Ifi203	0,000000	-2,69
ENSMUSG00000023206	Il15ra	0,000000	-2,72
ENSMUSG00000078920	Ifi47	0,000000	-2,73
ENSMUSG00000070427	Il18bp	0,000000	-2,73
ENSMUSG00000014602	Kif1a	0,000015	-2,73
ENSMUSG00000034422	Parp14	0,000000	-2,73
ENSMUSG00000024397	Aif1	0,000102	-2,74
ENSMUSG00000041827	Oasl1	0,000000	-2,74
ENSMUSG00000035208	Slfn8	0,000000	-2,75
ENSMUSG00000079507	H2-Q1	0,000000	-2,77
ENSMUSG00000062960	Kdr	0,000000	-2,77
ENSMUSG00000040247	Tbc1d10c	0,003712	-2,78
ENSMUSG00000035692	Isg15	0,000001	-2,78
ENSMUSG00000022357	Klhl38	0,000274	-2,79
ENSMUSG00000001281	Itgb7	0,000000	-2,79
ENSMUSG00000061232	H2-K1	0,000000	-2,80
ENSMUSG00000070501	Ifi214	0,000314	-2,81
ENSMUSG00000035929	H2-Q4	0,000000	-2,81
ENSMUSG00000059412	Fxyd2	0,000888	-2,82
ENSMUSG00000004609	Cd33	0,000000	-2,86
ENSMUSG00000021322	Aoah	0,000000	-2,91
ENSMUSG00000042333	Tnfrsf14	0,000004	-2,91
ENSMUSG00000041202	Pla2g2d	0,000982	-2,92
ENSMUSG00000034248	Slc25a37	0,000000	-2,92
ENSMUSG00000040483	Xaf1	0,000000	-2,92
ENSMUSG00000037321	Tap1	0,000000	-2,97
ENSMUSG00000034570	Inpp5j	0,000015	-3,01
ENSMUSG00000079419	Ms4a6c	0,000000	-3,01
ENSMUSG00000052563	D930048N14Rik	0,000000	-3,03
ENSMUSG00000048292	Olfr1417	0,004373	-3,04
ENSMUSG00000033227	Wnt6	0,001809	-3,06
ENSMUSG00000063234	Gpr84	0,000156	-3,07
ENSMUSG00000074361	C5ar2	0,000095	-3,12
ENSMUSG00000042474	Fcmr	0,003626	-3,14
ENSMUSG00000020638	Cmpk2	0,000000	-3,15
ENSMUSG00000073418	C4b	0,000000	-3,15
ENSMUSG00000035493	Tgfbi	0,000000	-3,16
ENSMUSG00000000794	Kcnn3	0,000314	-3,17
ENSMUSG00000014453	Blk	0,001016	-3,18
ENSMUSG00000030474	Siglece	0,000002	-3,21
ENSMUSG00000027322	Siglec1	0,000000	-3,23
ENSMUSG00000029822	Osbpl3	0,000000	-3,25
ENSMUSG00000041538	H2-Ob	0,001133	-3,26
ENSMUSG00000038264	Sema7a	0,000264	-3,29
ENSMUSG00000043263	Ifi209	0,000000	-3,35
ENSMUSG00000039196	Orm1	0,000000	-3,36

ENSMUSG00000040253	Gbp7	0,000000	-3,38
ENSMUSG00000028885	Smpdl3b	0,000000	-3,39
ENSMUSG00000067235	H2-Q10	0,000000	-3,40
ENSMUSG00000030109	Slc6a12	0,000000	-3,41
ENSMUSG00000024677	Ms4a6b	0,000000	-3,43
ENSMUSG00000036381	P2ry14	0,000000	-3,44
ENSMUSG00000045322	Tlr9	0,000000	-3,49
ENSMUSG00000028268	Gbp3	0,000000	-3,50
ENSMUSG00000074151	Nlrc5	0,000000	-3,50
ENSMUSG00000026822	Lcn2	0,000000	-3,50
ENSMUSG00000045826	Ptprcap	0,000306	-3,51
ENSMUSG00000030402	Ppm1n	0,002280	-3,52
ENSMUSG00000022534	Mefv	0,000000	-3,52
ENSMUSG00000018796	Acsl1	0,000000	-3,55
ENSMUSG00000060586	H2-Eb1	0,000000	-3,56
ENSMUSG00000000204	Slfn4	0,000000	-3,56
ENSMUSG00000050395	Tnfsf15	0,002233	-3,57
ENSMUSG00000050994	Adgb	0,000042	-3,58
ENSMUSG00000023341	Mx2	0,000002	-3,59
ENSMUSG00000037754	Ppp1r16b	0,000876	-3,66
ENSMUSG00000024334	H2-Oa	0,000016	-3,73
ENSMUSG00000037921	Ddx60	0,000000	-3,75
ENSMUSG00000045868	Gvin1	0,000000	-3,76
ENSMUSG00000031779	Ccl22	0,000000	-3,76
ENSMUSG00000031497	Tnfsf13b	0,000000	-3,77
ENSMUSG00000005672	Kit	0,000301	-3,79
ENSMUSG00000054072	Iigp1	0,000019	-3,85
ENSMUSG00000030107	Usp18	0,000000	-3,86
ENSMUSG00000034438	Gbp8	0,000267	-3,89
ENSMUSG00000075602	Ly6a	0,000002	-3,95
ENSMUSG00000067297	Ifit1bl2	0,001424	-4,00
ENSMUSG00000018168	Ikzf3	0,000054	-4,02
ENSMUSG00000029561	Oasl2	0,000000	-4,02
ENSMUSG00000031596	Slc7a2	0,000000	-4,04
ENSMUSG00000045502	Hcar2	0,000000	-4,06
ENSMUSG00000031722	Hp	0,000000	-4,08
ENSMUSG00000033022	Cdo1	0,000000	-4,09
ENSMUSG00000073421	H2-Ab1	0,000000	-4,19
ENSMUSG00000036594	H2-Aa	0,000000	-4,20
ENSMUSG00000030830	Itgal	0,000000	-4,20
ENSMUSG00000032323	Cyp11a1	0,003298	-4,23
ENSMUSG00000022587	Ly6e	0,000000	-4,28
ENSMUSG00000051998	Lax1	0,000480	-4,33
ENSMUSG00000028270	Gbp2	0,000000	-4,35
ENSMUSG00000046031	Fam26f	0,000001	-4,35
ENSMUSG00000047867	Gimap6	0,000004	-4,36
ENSMUSG00000099398	Ms4a14	0,000000	-4,38

ENSMUSG00000015314	Slamf6	0,000000	-4,42
ENSMUSG00000033355	Rtp4	0,000000	-4,49
ENSMUSG00000092517	Art2a-ps	0,001021	-4,50
ENSMUSG00000028071	Sh2d2a	0,000020	-4,50
ENSMUSG00000039264	Gimap3	0,000000	-4,54
ENSMUSG00000070661	Rnf186	0,000019	-4,57
ENSMUSG00000024399	Ltb	0,000004	-4,59
ENSMUSG00000033825	Tpsb2	0,000203	-4,60
ENSMUSG00000022504	Ciita	0,000000	-4,62
ENSMUSG00000079017	Ifi27l2a	0,000000	-4,65
ENSMUSG00000022899	Slc15a2	0,000011	-4,78
ENSMUSG00000091405	Hist2h4	0,000011	-4,80
ENSMUSG00000105504	Gbp5	0,000000	-4,81
ENSMUSG00000079018	Ly6c1	0,002544	-4,93
ENSMUSG00000064262	Gimap8	0,000001	-5,00
ENSMUSG00000055413	H2-Q5	0,000000	-5,01
ENSMUSG00000079363	Gbp4	0,000000	-5,03
ENSMUSG00000074345	Tnfaip8l3	0,000068	-5,06
ENSMUSG00000039304	Tnfsf10	0,000027	-5,12
ENSMUSG00000078922	Tgtp1	0,000000	-5,27
ENSMUSG00000001865	Cpa3	0,000587	-5,32
ENSMUSG00000009292	Trpm2	0,000000	-5,36
ENSMUSG00000032661	Oas3	0,000000	-5,36
ENSMUSG00000016283	H2-M2	0,000000	-5,41
ENSMUSG00000032915	Adgre4	0,000046	-5,48
ENSMUSG00000038390	Gpr162	0,000000	-5,54
ENSMUSG00000022225	Cma1	0,000272	-5,67
ENSMUSG00000034459	Ifit1	0,000000	-5,69
ENSMUSG00000024610	Cd74	0,000000	-5,92
ENSMUSG00000027514	Zbp1	0,000000	-6,02
ENSMUSG00000068129	Cst7	0,000000	-6,06
ENSMUSG00000064246	Chil1	0,000029	-6,22
ENSMUSG00000021200	Asb2	0,000020	-6,23
ENSMUSG00000051726	Kcnf1	0,000065	-6,25
ENSMUSG00000027347	Rasgrp1	0,000000	-6,25
ENSMUSG00000056290	Ms4a4b	0,000000	-6,50
ENSMUSG00000028965	Tnfrsf9	0,000005	-6,64
ENSMUSG00000078921	Tgtp2	0,000000	-6,68
ENSMUSG00000036523	Greb1	0,000113	-6,77
ENSMUSG00000024675	Ms4a4c	0,000000	-6,88
ENSMUSG00000000386	Mx1	0,000000	-6,90
ENSMUSG00000045932	Ifit2	0,000000	-6,96
ENSMUSG00000078616	Trim30c	0,000000	-7,06
ENSMUSG00000104876	Trdc	0,000000	-7,21
ENSMUSG00000025498	Irf7	0,000000	-7,22
ENSMUSG00000032690	Oas2	0,000000	-7,45
ENSMUSG00000104713	Gbp6	0,000000	-7,84

ENSMUSG00000074896	Ifit3	0,000000	-8,11
ENSMUSG00000062488	Ifit3b	0,000000	-8,14
ENSMUSG00000020010	Vnn3	0,000000	-8,36
ENSMUSG00000105096	Gbp10	0,000000	-8,43
ENSMUSG00000076490	Trbc1	0,000000	-8,79
ENSMUSG00000024210	Ip6k3	0,000011	-9,13
ENSMUSG00000068227	Il2rb	0,000000	-10,23
ENSMUSG00000060550	H2-Q7	0,000000	-10,26
ENSMUSG00000073409	H2-Q6	0,000000	-10,80
ENSMUSG00000025461	Cd163l1	0,000000	-12,32
ENSMUSG00000028037	Ifi44	0,000000	-12,61
ENSMUSG00000066677	Ifi208	0,000000	-12,83
ENSMUSG00000035042	Ccl5	0,000000	-12,99
ENSMUSG00000037849	Ifi206	0,000000	-13,19
ENSMUSG00000078763	Slfn1	0,000000	-14,01
ENSMUSG00000029417	Cxcl9	0,000000	-15,10
ENSMUSG00000020826	Nos2	0,000000	-23,50
ENSMUSG00000022584	Ly6c2	0,000000	-25,27
ENSMUSG00000073491	Ifi213	0,000000	-26,38
ENSMUSG00000022586	Ly6i	0,000000	-33,67
ENSMUSG00000079186	Gzmc	0,000000	-38,75
ENSMUSG00000015437	Gzmb	0,000000	-39,00
