# Supplementary material for: Tissue-infiltrating macrophages mediate an exosome-based metabolic reprogramming upon DNA damage
Source: Nat Commun. 2020 Jan 2;11:42. doi: 10.1038/s41467-019-13894-9 (PMC6940362; doi:10.1038/s41467-019-13894-9)
Supplement: Supplementary file 5 — Supplementary data 2 [file 41467_2019_13894_MOESM5_ESM.pdf]

Supplementary data 2. Overview of the 211 proteins identified in Er1 F/- macrophage media compared to Er1 F/+ macrophage controls

	% coverage				No of peptides				Signal intensity			
UniProt	Er1f/+ 1	Er1f/+ 2	Er1f/- 1	Er1f/- 2	Er1f/+ 1	Er1f/+ 2	Er1f/- 1	Er1f/- 2	Er1f/+ 1	Er1f/+ 2	Er1f/- 1	Er1f/- 2
Q6PI17	0%	0%	100%	100%	0	0	4	5	1	1	1200000	519000
A2APX3	0%	0%	100%	66%	0	0	2	1	1	1	574000	128000
P10605	100%	95%	100%	100%	23	1	19	23	9550000	385000	9580000	1,5E+07
P97426	0%	0%	100%	100%	0	0	2	2	1	1	240000	54900
P08071	100%	100%	100%	100%	7	17	17	25	1380000	2950000	3720000	4480000
P97821	100%	99%	100%	100%	3	0	2	6	177000	1	854000	165000
O88968	100%	0%	100%	100%	7	0	7	10	269000	1	1220000	229000
Q60648	100%	0%	100%	100%	2	0	6	5	259000	1	1450000	1230000
Q61646	99%	8%	100%	100%	2	0	2	2	46500	1	481000	70800
G3X8Q5	0%	11%	100%	100%	0	0	1	3	1	1	553000	510000
O08692	82%	0%	100%	100%	1	0	3	4	75100	1	745000	446000
P50429	100%	0%	98%	100%	2	0	1	1	44700	1	121000	47300
O35744	100%	88%	100%	100%	24	0	24	22	5260000	1	7820000	4750000
P11672	100%	0%	100%	100%	5	0	8	5	549000	1	3880000	5820000
P32261	0%	50%	100%	100%	0	1	2	2	1	59100	667000	731000
P20060	100%	99%	100%	100%	7	0	4	3	582000	1	446000	488000
G3X8T3	100%	50%	100%	100%	7	1	6	5	671000	25300	2660000	2760000
Q8BVU5	0%	66%	65%	100%	0	0	1	1	1	1	119000	14500
O09159	100%	97%	100%	100%	11	0	6	11	2560000	1	3240000	2290000
P18242	100%	100%	100%	100%	22	4	21	19	9240000	263000	8540000	1E+07
P24369	0%	0%	76%	100%	0	0	1	1	1	1	108000	73800
O35855	100%	64%	100%	100%	6	0	2	7	671000	1	520000	955000
Q9Z0S1	100%	0%	100%	100%	3	0	3	6	244000	1	433000	633000
O88587	22%	0%	71%	100%	0	0	1	1	1	1	93600	19700
P61027	0%	0%	100%	100%	0	0	2	3	1	1	1160000	313000
D3YYD5	0%	0%	100%	100%	0	0	2	3	1	1	525000	169000
P51150	100%	0%	100%	100%	2	0	7	7	268000	1	1660000	427000
Q9DCD0	100%	94%	100%	100%	40	1	38	33	9240000	1240000	1,1E+07	1,4E+07
B1AWZ5	0%	0%	98%	99%	0	0	1	1	1	1	90600	2789
P97807	100%	67%	100%	100%	11	0	13	9	905000	1	1460000	1240000
A6PWC3	0%	72%	77%	100%	0	0	1	5	1	1	46400	540000
Q5XJY5	100%	100%	100%	100%	5	1	7	18	763000	37900	1240000	1570000
Q8QZT1	100%	0%	100%	100%	1	0	2	2	58300	1	377000	85700
P47738	100%	65%	100%	100%	23	1	22	20	3150000	7530	3240000	2800000
Q8VEH3	0%	0%	100%	100%	0	0	1	2	1	1	180000	172000
Q8BWT1	100%	54%	100%	72%	5	0	3	1	88000	1	67300	86100
Q9D892	0%	0%	100%	100%	0	0	6	5	1	1	1380000	1710000
H7BWY6	0%	0%	76%	99%	0	0	1	1	1	1	88000	465000
P47791	100%	100%	100%	100%	11	2	9	18	1380000	81700	1180000	2140000
P35278	100%	55%	100%	100%	3	1	5	7	640000	86100	1040000	921000
Q91Z53	100%	85%	100%	100%	4	0	2	6	482000	1	491000	543000
Q8K2Q0	100%	0%	76%	98%	2	0	1	1	147000	1	368000	505000
Q9D154	100%	87%	100%	100%	19	1	14	19	2580000	53800	4340000	3700000
P23591	100%	73%	100%	100%	9	0	5	8	705000	1	901000	807000
Q3TYS2	0%	0%	76%	98%	0	0	1	1	1	1	77300	65500
Q9WTL7	100%	0%	100%	100%	1	0	2	6	72400	1	122000	276000
P16460	100%	0%	100%	100%	14	0	7	11	193000	1	398000	344000
P11352	0%	0%	100%	100%	0	0	2	2	1	1	165000	44500
Q60854	100%	79%	100%	100%	23	0	18	17	897000	1	814000	926000
Q63829	0%	0%	100%	100%	0	0	3	2	1	1	578000	125000
Q7TNV0	100%	88%	100%	100%	1	0	2	2	82500	1	107000	193000
Q921H8	100%	94%	100%	100%	8	1	7	16	781000	123000	1060000	788000
P28063	100%	0%	100%	100%	9	0	8	10	1920000	1	4470000	4640000
P45377	100%	58%	100%	100%	3	0	2	2	327000	1	284000	403000
Q8BTZ7	100%	0%	100%	100%	2	0	2	6	206000	1	382000	263000
P40336	100%	81%	100%	100%	7	0	3	10	617000	1	861000	985000
P60229	99%	0%	100%	100%	0	0	4	2	1	1	239000	210000
Q9CZ30	100%	0%	100%	100%	3	0	6	5	347000	1	782000	388000
P84096	21%	0%	100%	100%	0	0	5	4	1	1	1450000	1180000
O08807	100%	0%	98%	100%	1	0	1	3	530000	1	4660000	1,1E+07
P35279	0%	0%	100%	100%	0	0	3	0	1	1	1030000	77100
Q9CX00	0%	0%	100%	100%	0	0	5	6	1	1	1480000	735000
Q99LX0	100%	0%	100%	100%	4	0	8	9	376000	1	3850000	2260000

Q3TLP8	0%	0%	100%	100%	0	0	5	7	1	1	2800000	1350000
P60766	0%	79%	100%	100%	0	0	3	5	1	1	1950000	5060000
P03958	100%	0%	100%	100%	1	0	1	3	32500	1	156000	236000
A2AU52	0%	89%	100%	66%	0	0	2	1	1	1	271000	154000
Q505F5	100%	98%	100%	100%	2	1	3	4	42700	14600	131000	82900
Q8K183	100%	0%	100%	100%	16	0	11	15	1700000	1	5840000	1620000
P61089	0%	0%	100%	100%	0	0	5	4	1	1	1950000	508000
Q9CQM8	0%	0%	100%	100%	0	0	3	3	1	1	445000	309000
P97315	0%	0%	100%	100%	0	0	2	3	1	1	257000	198000
Q8CHP8	100%	0%	100%	100%	6	0	4	8	312000	1	725000	643000
O55234	0%	0%	100%	100%	0	0	2	2	1	1	441000	80400
Q9Z1Q9	100%	100%	100%	100%	13	6	11	21	3620000	766000	4190000	3480000
Q80X41	52%	78%	100%	100%	0	0	1	1	1	1	39300	41300
D3Z1H9	82%	60%	100%	100%	1	0	3	4	89500	1	939000	460000
P53994	0%	0%	99%	100%	0	0	1	2	1	1	143000	61800
Q99JI6	0%	0%	98%	100%	0	0	1	5	1	1	547000	973000
D3Z158	100%	50%	100%	100%	2	1	2	6	157000	16500	338000	362000
Q9Z2W0	100%	56%	100%	100%	7	0	6	9	431000	1	350000	498000
Q9R0N0	100%	86%	100%	100%	3	0	3	6	234000	1	447000	331000
Q61598	100%	100%	100%	100%	31	1	30	34	6130000	37000	6240000	7450000
Q9CX34	100%	0%	100%	100%	2	0	2	5	205000	1	527000	195000
B1AQF4	0%	0%	100%	100%	0	0	4	4	1	1	1440000	323000
P62821	100%	0%	100%	100%	10	0	12	11	1360000	1	3540000	1430000
Q3B7Z2	0%	99%	100%	98%	0	1	2	1	1	12100	255000	39500
Q8BVE3	100%	0%	100%	100%	5	0	4	5	265000	1	411000	336000
O55135	80%	92%	99%	100%	1	0	1	5	185000	1	149000	247000
Q3THS6	60%	0%	100%	100%	1	0	2	3	93100	1	173000	209000
P30681	100%	100%	100%	100%	20	2	19	32	6930000	7430000	1,1E+07	1,3E+07
Q9D8B3	100%	74%	77%	100%	3	0	1	4	154000	1	292000	274000
Q68FH4	100%	81%	100%	100%	1	0	4	4	110000	1	248000	173000
Q64520	0%	0%	100%	100%	0	0	3	3	1	1	547000	195000
D3Z7C6	46%	0%	100%	98%	0	0	2	1	1	1	361000	494000
Q9D8N0	100%	81%	100%	100%	17	0	16	15	1770000	1	2510000	4090000
Q9CXY6	100%	0%	70%	100%	3	0	2	4	57900	1	95700	92500
P28474	100%	73%	100%	100%	8	0	3	9	811000	1	1450000	1510000
A2ADY9	0%	67%	100%	63%	0	0	2	1	1	1	104000	96500
O35593	100%	57%	98%	100%	2	0	1	5	76600	1	117000	102000
E0CXA9	82%	0%	71%	100%	1	0	1	2	5606	1	16600	9490
Q61096	98%	100%	100%	100%	0	1	2	2	1	145000	122000	176000
Q9QZ08	100%	0%	77%	100%	2	0	1	8	101000	1	185000	404000
G5E8N7	100%	100%	100%	100%	2	4	5	8	216000	293000	617000	640000
Q99JI4	100%	67%	100%	100%	14	0	9	10	341000	1	337000	439000
O54879	48%	0%	100%	100%	0	0	2	2	1	1	360000	471000
Q91YI0	100%	0%	100%	100%	7	0	8	7	514000	1	1070000	814000
Q99JY9	100%	50%	100%	100%	26	1	23	25	6410000	1401	6430000	7890000
J3QJZ3	82%	0%	100%	100%	1	0	5	3	29800	1	1590000	249000
Q9CSU0	100%	0%	77%	100%	2	0	1	7	87500	1	160000	221000
P08030	100%	0%	100%	100%	3	0	5	6	474000	1	3240000	1200000
Q8VBV7	0%	51%	100%	99%	0	0	3	1	1	1	501000	28400
E9QAZ2	100%	65%	100%	100%	2	0	3	4	488000	1	1290000	612000
Q9WVA3	100%	93%	100%	100%	4	0	2	8	235000	1	306000	458000
Q64727	100%	100%	100%	100%	20	8	12	45	5390000	757000	4830000	8230000
Q61171	19%	0%	100%	100%	0	0	11	10	1	1	8740000	8600000
Q5SUR0	100%	13%	100%	100%	2	0	3	7	660000	1	1250000	893000
P62827	100%	91%	100%	100%	9	0	11	13	5540000	1	6740000	5400000
Q99KQ4	100%	0%	100%	100%	10	0	11	17	614000	1	1220000	1580000
Q91Z50	100%	0%	100%	100%	0	0	1	2	1	1	120000	167000
P46664	100%	0%	100%	100%	3	0	9	7	235000	1	962000	664000
Q91V55	100%	94%	100%	100%	8	1	9	10	873000	2290000	2670000	5260000
P63085	100%	83%	100%	100%	6	0	6	10	1890000	1	2530000	2990000
Q9WVJ2	100%	0%	100%	100%	11	0	5	7	368000	1	705000	333000
P16110	100%	95%	100%	100%	11	1	11	12	6920000	414000	7460000	8050000
Q07813	0%	0%	100%	100%	0	0	5	7	1	1	1230000	412000
Q8BK67	100%	0%	100%	100%	1	0	5	4	60400	1	411000	306000
P70296	82%	0%	100%	100%	1	0	5	5	267000	1	1700000	3190000
P53996	0%	0%	100%	99%	0	0	2	1	1	1	172000	499000
P00920	68%	0%	69%	100%	1	0	1	2	13100	1	24800	10500
Q9CR16	100%	72%	100%	100%	11	0	5	17	760000	1	1270000	1680000

A2A6J7	98%	79%	100%	100%	1	0	2	2	43600	1	138000	179000
P61759	0%	0%	76%	100%	0	0	1	3	1	1	127000	82600
Q61792	100%	100%	100%	100%	7	3	4	7	903000	288000	1510000	1610000
P63260	98%	90%	100%	72%	1	1	2	1	9,8E+07	2,1E+07	1E+08	1,1E+08
P41241	100%	0%	100%	100%	3	0	4	2	142000	1	297000	130000
O88543	100%	69%	98%	100%	4	0	1	4	220000	1	304000	353000
Q05144	0%	0%	100%	100%	0	0	5	4	1	1	3860000	2610000
F6YTZ4	99%	0%	100%	100%	1	0	2	4	40600	1	137000	137000
Q91YR1	100%	0%	100%	100%	7	0	7	12	612000	1	2030000	1000000
E9Q1G8	100%	49%	100%	100%	8	0	8	6	811000	1	680000	765000
Q7M6Y3	100%	72%	100%	100%	6	0	4	11	525000	1	939000	522000
Q9JLJ2	100%	0%	100%	100%	5	0	6	8	365000	1	588000	896000
P09602	0%	0%	76%	100%	0	0	1	2	1	1	64200	387000
Q9CZU3	16%	0%	77%	100%	0	0	1	2	1	1	10200	174000
Q9Z0N1	100%	0%	100%	100%	5	0	6	7	470000	1	1190000	1560000
Q9CWZ3	82%	0%	76%	100%	1	0	1	2	38500	1	263000	102000
P35550	99%	0%	69%	100%	1	0	1	2	48400	1	193000	47600
P70670	100%	70%	100%	100%	3	0	2	7	371000	1	285000	297000
Q8VDJ3	100%	89%	86%	100%	2	0	1	4	480000	1	1190000	441000
Q9D1P4	100%	70%	77%	100%	5	0	1	6	217000	1	247000	271000
Q6ZWN5	100%	0%	100%	100%	2	0	9	9	137000	1	3100000	1620000
Q76MZ3	100%	100%	100%	100%	25	20	29	24	3060000	3410000	5040000	5140000
Q61166	65%	0%	100%	100%	0	0	2	4	1	1	120000	105000
Q9QZE7	100%	0%	100%	100%	2	0	2	4	18800	1	31500	35800
Q9WVA4	43%	21%	100%	100%	0	0	6	15	1	1	1710000	2340000
P47915	0%	95%	100%	100%	0	0	3	5	1	1	1060000	1640000
Q571I9	62%	77%	100%	100%	1	0	3	3	12400	1	362000	166000
Q8BMJ3	0%	66%	100%	100%	0	0	2	5	1	1	432000	1640000
Q9Z2M7	99%	52%	100%	100%	1	0	2	4	61900	1	162000	54900
O09106	95%	100%	99%	100%	0	2	1	2	1	153000	345000	161000
P35821	58%	80%	65%	100%	1	0	1	2	31100	1	168000	84800
Q9JJ28	100%	50%	100%	100%	4	1	3	9	776000	25100	845000	1130000
O88844	100%	0%	100%	100%	24	0	30	32	3880000	1	6520000	8200000
Q08024	0%	0%	100%	100%	0	0	3	3	1	1	632000	119000
P35492	68%	50%	100%	100%	1	1	3	2	36400	12600	307000	71200
P31725	56%	39%	71%	100%	0	0	1	2	1	1	192000	249000
Q9CQF3	77%	0%	100%	100%	0	0	1	1	1	1	30400	15300
G3X956	100%	0%	100%	100%	2	0	2	7	432000	1	949000	869000
Q64514	100%	100%	100%	100%	8	4	7	20	1880000	285000	2580000	3430000
Q9R1P4	100%	70%	100%	100%	13	0	8	16	1450000	1	1480000	2180000
P46471	100%	70%	100%	100%	4	0	8	6	225000	1	428000	452000
Q9ESP1	78%	0%	100%	100%	0	0	2	5	1	1	541000	513000
P60843	100%	0%	100%	100%	17	0	18	12	1720000	1	3000000	2630000
P54923	100%	0%	100%	100%	2	0	3	3	140000	1	599000	182000
Q8CIH5	100%	100%	100%	100%	15	4	14	27	4900000	836000	6310000	4940000
Q9DAR7	100%	0%	100%	100%	4	0	3	8	250000	1	1080000	293000
P43276	100%	69%	100%	100%	7	0	5	12	2250000	1	1760000	1750000
Q61599	100%	0%	100%	100%	4	0	11	12	798000	1	3030000	1690000
Q5FWK3	100%	77%	100%	100%	3	0	4	4	273000	1	306000	427000
P49443	96%	0%	65%	100%	0	0	1	5	1	1	101000	197000
Q3UM45	100%	0%	100%	100%	6	0	3	5	186000	1	434000	178000
P61161	100%	87%	100%	100%	27	0	19	24	4400000	1	7160000	4580000
P23198	0%	54%	100%	100%	0	0	5	11	1	1	1510000	2680000
Q921Z5	0%	0%	100%	66%	0	0	2	1	1	1	248000	25400
P70372	80%	0%	100%	100%	1	0	2	3	314000	1	472000	290000
Q9WTM5	100%	69%	100%	100%	4	0	5	4	403000	1	425000	324000
Q91VC3	60%	0%	100%	100%	1	0	2	4	120000	1	605000	756000
Q6P069	0%	0%	76%	100%	0	0	1	2	1	1	215000	372000
Q920E5	100%	49%	100%	100%	14	0	7	16	2310000	1	4100000	2340000
Q99L27	99%	0%	65%	100%	0	0	1	4	1	1	117000	245000
Q7TQI3	100%	0%	77%	100%	1	0	1	4	120000	1	401000	489000
P47811	100%	0%	100%	100%	10	0	7	16	927000	1	1450000	2210000
Q9DBC7	100%	0%	100%	100%	7	0	3	3	106000	1	242000	206000
Q6ZWZ7	0%	0%	100%	100%	0	0	3	2	1	1	414000	802000
P61164	100%	0%	100%	100%	4	0	5	5	341000	1	581000	294000
Q3UMB9	100%	0%	100%	100%	1	0	2	3	69200	1	216000	246000
Q9QZB9	0%	0%	99%	66%	0	0	1	1	1	1	65200	22500
Q6WVG3	100%	0%	100%	100%	3	0	3	10	275000	1	887000	1010000

Q61029	100%	0%	77%	100%	2	0	1	8	173000	1	261000	322000
Q99KH8	100%	0%	65%	100%	2	0	1	6	54000	1	82800	308000
Q99020	100%	0%	100%	100%	15	0	5	17	707000	1	1300000	1320000
P62715	56%	0%	99%	100%	0	0	1	6	1	1	51100	166000
Q8VHM5	99%	99%	73%	100%	1	1	1	2	153000	16300	137000	170000
Q8C1B7	100%	74%	100%	100%	4	0	2	2	670000	1	605000	739000
P62334	100%	49%	100%	100%	11	0	8	7	311000	1	282000	285000
Q9D8U8	100%	90%	100%	100%	9	0	11	9	1950000	1	1690000	2000000
P51410	49%	75%	100%	100%	0	0	2	1	1	1	469000	54600
O35381	100%	20%	100%	100%	5	0	4	6	665000	1	878000	532000
Q8BYC6	0%	0%	77%	100%	0	0	1	3	1	1	239000	272000
P27659	100%	0%	100%	100%	6	0	6	4	77300	1	118000	71300
