# Supplementary material for: Tissue-infiltrating macrophages mediate an exosome-based metabolic reprogramming upon DNA damage
Source: Nat Commun. 2020 Jan 2;11:42. doi: 10.1038/s41467-019-13894-9 (PMC6940362; doi:10.1038/s41467-019-13894-9)
Supplement: Supplementary file 10 — Reporting Summary [file 41467_2019_13894_MOESM10_ESM.pdf]

nature research

Corresponding author(s): *Double-blind peer review submissions: write DBPR and your manuscript number here instead of author names.*

Last updated by author(s): Aug 19, 2019

Reporting Summary

Nature Research wishes to improve the reproducibility of the work that we publish. This form provides structure for consistency and transparency in reporting. For further information on Nature Research policies, seeAuthors & Referees and theEditorial Policy Checklist .

Statistics

For all statistical analyses, confirm that the following items are present in the figure legend, table legend, main text, or Methods section.

n/a	Confirmed	
☐	☒	The exact sample size (*n*) for each experimental group/condition, given as a discrete number and unit of measurement
☐	☒	A statement on whether measurements were taken from distinct samples or whether the same sample was measured repeatedly
☐	☒	The statistical test(s) used AND whether they are one- or two-sided<br>*Only common tests should be described solely by name; describe more complex techniques in the Methods section.*
☒	☐	A description of all covariates tested
☒	☐	A description of any assumptions or corrections, such as tests of normality and adjustment for multiple comparisons
☒	☐	A full description of the statistical parameters including central tendency (e.g. means) or other basic estimates (e.g. regression coefficient) AND variation (e.g. standard deviation) or associated estimates of uncertainty (e.g. confidence intervals)
☐	☒	For null hypothesis testing, the test statistic (e.g. *F*, *t*, *r*) with confidence intervals, effect sizes, degrees of freedom and *P* value noted<br>*Give P values as exact values whenever suitable.*
☒	☐	For Bayesian analysis, information on the choice of priors and Markov chain Monte Carlo settings
☒	☐	For hierarchical and complex designs, identification of the appropriate level for tests and full reporting of outcomes
☒	☐	Estimates of effect sizes (e.g. Cohen's *d*, Pearson's *r*), indicating how they were calculated

Our web collection on statistics for biologists contains articles on many of the points above.

Software and code

Policy information about availability of computer code

Data collection: *Provide a description of all commercial, open source and custom code used to collect the data in this study, specifying the version used OR state that no software was used.*

Data analysis: *Provide a description of all commercial, open source and custom code used to analyse the data in this study, specifying the version used OR state that no software was used.*

For manuscripts utilizing custom algorithms or software that are central to the research but not yet described in published literature, software must be made available to editors/reviewers. We strongly encourage code deposition in a community repository (e.g. GitHub). See the Nature Research guidelines for submitting code & software for further information.

Data

Policy information about availability of data

All manuscripts must include a data availability statement. This statement should provide the following information, where applicable:

- Accession codes, unique identifiers, or web links for publicly available datasets
- A list of figures that have associated raw data
- A description of any restrictions on data availability

All data generated during this study are included in the published article (and its supplementary information files)

Field-specific reporting

Please select the one below that is the best fit for your research. If you are not sure, read the appropriate sections before making your selection.

☒ Life sciences ☐ Behavioural & social sciences ☐ Ecological, evolutionary & environmental sciences

October 2018

Life sciences study design

All studies must disclose on these points even when the disclosure is negative.

Sample size	Sample size calculation was based on 1. effect size (the difference between the mean of two groups), 2. the standard deviation or standard error of the mean variability within the sample and 3. the decision of direction of effect (two tailed for all experiments performed), 4. expected death of animals.
Data exclusions	No data were excluded from the analysis.
Replication	All experiments were reproduced at least 3 times (biological replicates) and in multiple technical replicates (at least 3 technical replicates).
Randomization	Samples were allocated to experimental groups according to genotypes or treatments.
Blinding	Investigators were not blinded to group allocations for all experiments performed in this work. This is due to the fact that the animal models used in this work have obvious phenotypes.

Reporting for specific materials, systems and methods

We require information from authors about some types of materials, experimental systems and methods used in many studies. Here, indicate whether each material, system or method listed is relevant to your study. If you are not sure if a list item applies to your research, read the appropriate section before selecting a response.

Materials & experimental systems

n/a	Involved in the study	
☐	☒	Antibodies
☐	☒	Eukaryotic cell lines
☒	☐	Palaeontology
☐	☒	Animals and other organisms
☒	☐	Human research participants
☒	☐	Clinical data

Methods

n/a	Involved in the study	
☒	☐	ChIP-seq
☐	☒	Flow cytometry
☒	☐	MRI-based neuroimaging

Antibodies

Antibodies used	Antibodies against Rac2 (C-11, WB: 1:500, IF: 1:100), RhoA (26C4, WB: 1:500, IF: 1/100), CD9 (C-4, WB: 1:500, IF: 1/100), Glut2 (H-67, WB: 1:500, IF: 1:100), Glut3 (B-6, WB: 1:500, IF: 1:100), LC3 (C-9, WB: 1:500, IF: 1:500), FancI (H102, IF: 1:50), Ercc1 (D-10, WB:1:500, IF: 1:50), Albumin (P-20, WB: 1:500, IF: 1/200), Amylase (G-10, WB: 1:500, IF:1:100), biotin (Rockland, 600-401-098, IF:1:500), LaminB1 (ab16048, WB:1:1000), p62 (SQSTM1, MBL PM045, WB:1:5000, IF:1:1000), goat anti-rat IgG-CFL 647 (sc-362293, IF: 1:1000) and donkey anti-goat IgG-HRP (sc-2020, WB: 1:5000) were from SantaCruz Biotechnology. γH2AX (05-636, IF: 1:12000), pATM (05-740, IF: 1:100), Rad51 (ABE257, IF: 1:100), Caspase3 (AB3623, IF: 1/200), p-Glut (Ser226) (ABN991), Goat anti-Rabbit IgG Antibody, Peroxidase Conjugated (AP132P, WB: 1:10000) and Goat Anti-Mouse IgG Antibody, Peroxidase Conjugated, H+L (AP124P, WB: 1:10000) were from Millipore. GM130 (clone 35, wb: 1:500, IF: 1:200) was from BD Transduction Laboratories. Glut1 (ab40084, WB:1:300, IF: 1:150), Calreticulin (ab2907, IF:1:500), Grp78/BiP (ab21685, WB: 1:500, IF: 1;200), F4/80 (ab6640, WB:1:500), b-tubulin (ab6046, WB:1:1000) and iNOS (ab15323, WB:1:500, IF: 1:100) were from Abcam. Alix (#2171, WB: 1:500), Rab10 (#8127, WB: 1:500, IF: 1:100), NF-κB p65 (#8242, IF: 1:100), p4EBP1 (#2855, WB: 1:500) and Phospho-p70 S6 Kinase (Ser371) (#9208, WB: 1:500) were from Cell Signaling Technology. VCAM (P8B1, IF: 1:200), CD45 (H5A5, IF: 1:200), ICAM (P2A4, IF: 1:100), PECAM (2H8, IF: 1:200) and Mac1 (M1/70.15.11.5.2, IF: 1:200) were from Developmental Studies Hybridoma Bank (DSHB). Rac1 (ARC03, WB:1:500, IF: 1:50) was from Cytoskeleton. Goat anti-Mouse IgG (H+L) Cross-Adsorbed Secondary Antibody, Alexa Fluor 488 (A-11001, IF: 1:2000), Goat anti-Mouse IgG (H+L) Cross-Adsorbed Secondary Antibody, Alexa Fluor 555 (A-21422, IF:1:2000), Donkey anti-Rabbit IgG (H+L) Highly Cross-Adsorbed Secondary Antibody, Alexa Fluor 488 (A-21206, IF:1:2000), Donkey anti-Rabbit IgG (H+L) Highly Cross-Adsorbed Secondary Antibody, Alexa Fluor 555 (A-31572, IF:1:2000), Goat anti-Rat IgG (H+L) Cross-Adsorbed Secondary Antibody, Alexa Fluor 555 (A-21434, IF:1:2000) and DAPI (62247, IF:1:20000) were from ThermoFisher.
Validation	The validation of all primary antibodies was based on available references in the manuscript and information provided on the manufacturer website and associated literature.

Eukaryotic cell lines

Policy information about cell lines

Cell line source(s)	All primary cells were derived from Er1F/+ or Er1F/- animal models (as indicated in the manuscript).

Authentication	The genotype of primary cells was authenticated with PCR, sequencing, western blotting and/or confocal imaging for the expression of ERCC1
Mycoplasma contamination	All primary cells were tested negative for mycoplasma.
Commonly misidentified lines (See ICLAC register)	We have not used any misidentified lines in this manuscript.

Animals and other organisms

Policy information about studies involving animals; ARRIVE guidelines recommended for reporting animal research

Laboratory animals	Mus musculus; strain: Bl/6; sex: males or females (as indicated).
Wild animals	There was no use of wild animals.
Field-collected samples	There is no work with field-collected samples.
Ethics oversight	Foundation of Research and Technology Hellas.

Note that full information on the approval of the study protocol must also be provided in the manuscript.

Flow Cytometry

Plots

Confirm that:

- [x] The axis labels state the marker and fluorochrome used (e.g. CD4-FITC).
- [x] The axis scales are clearly visible. Include numbers along axes only for bottom left plot of group (a 'group' is an analysis of identical markers).
- [x] All plots are contour plots with outliers or pseudocolor plots.
- [x] A numerical value for number of cells or percentage (with statistics) is provided.

Methodology

Sample preparation	Pancreas and spleen were minced in 1xPBS/1%BSA/0.1%NaN3 and further processed with a Dounce homogenizer. Red blood cells were lysed in ice cold red blood cell lysis buffer. Homogenized tissue was further washed in PBS-BSA buffer and passed through a 100μM wire mesh. Peripheral blood was isolated with heart puncture and bone marrow was isolated from femurs and tibias. Erythrocytes were lysed as previously mentioned. Samples were further washed in PBS/BSA
Instrument	FACS Calibur (BD Biosciences)
Software	We collected and analyzed the data using FlowJo 7.6.5 software
Cell population abundance	No sorting was performed with the flow-cytometer.
Gating strategy	To gate samples for FACS analysis, cells were initially gated by Forward scatter vs.side scatter to separate cell events from debris. Then a CD45+ CD11b+.gate was set to evaluate the number of the CD45+ CD11b+.population. In this case the fluorescence pattern of unstained cells (background fluorescence) and single stained cells was used to set the parameters of the double gate.

- [ ] Tick this box to confirm that a figure exemplifying the gating strategy is provided in the Supplementary Information.
